# Network divergence analysis identifies adaptive gene modules and two orthogonal vulnerability axes in pancreatic cancer

**DOI:** 10.1002/1878-0261.70218

**Published:** 2026-03-01

**Authors:** Brian Nelson, Lyanne Delgado‐Coka, Natalia Marchenko, Luisa F. Escobar‐Hoyos, Kenneth R. Shroyer, Alisa Yurovsky, Trey Ideker, Gábor Balázsi, Thomas MacCarthy, Scott Powers

**Affiliations:** ^1^ Department of Applied Mathematics and Statistics Stony Brook University New York NY USA; ^2^ Department of Pathology Stony Brook University New York NY USA; ^3^ Department of Therapeutic Radiology Yale University New Haven CT USA; ^4^ Department of Biomedical Informatics Stony Brook University New York NY USA; ^5^ Department of Medicine University of California, San Diego La Jolla CA USA; ^6^ Department of Biomedical Engineering Stony Brook University New York NY USA; ^7^ Louis and Beatrice Laufer Center for Physical and Quantitative Biology Stony Brook University Stony Brook NY USA; ^8^ Stony Brook Cancer Center Stony Brook University Stony Brook NY USA

**Keywords:** cell‐state plasticity, functional genomics, gene regulatory networks, pancreatic ductal adenocarcinoma, single‐cell transcriptomics, therapeutic vulnerabilities

## Abstract

Transcriptional heterogeneity in pancreatic ductal adenocarcinoma (PDAC) arises not only from changes in gene expression but also from dynamic rewiring of gene–gene coordination. Using a divergent‐edge framework applied to 77 155 malignant cells from 42 tumors, we identified four reproducible adaptive modules—integrated growth‐energy (IGE), stress‐adaptive transcription (SAT), IL‐2‐linked immune evasion (IL2), and multi‐pathway collective invasion (MPC)—that cut across canonical PDAC states and reflect distinct regulatory programs. Integrating these modules with CRISPR–Cas9 dependency profiles and PRISM drug‐response data revealed that adaptive behaviors collapse into two higher‐order axes: a biosynthetic–metabolic IGE axis enriched for translational and DNA‐repair dependencies, and a broader SAT–IL2–MPC stress–immune–invasion axis characterized by proteostasis, cytokine‐linked, and cytoskeletal vulnerabilities. This architecture emerges only when divergent‐edge modules are mapped into functional genomics space. Module activity also carried clinical relevance in PDAC. SAT‐high tumors showed poorer survival, while MPC‐high tumors exhibited a similar adverse trend; together, these modules defined a stress–immune–invasion poor‐prognosis axis. In contrast, IGE activity showed no overall risk association, although an optimal‐cut point–defined IGE‐high subgroup displayed modestly improved survival.

## Graphical abstract

Tumors contain diverse cellular states whose behavior is shaped by context‐dependent gene coordination. By comparing gene–gene relationships across biological contexts, we identify adaptive transcriptional modules that reorganize into distinct vulnerability axes. Integrating these modules with functional and clinical data reveals how context‐dependent regulation structures tumor behavior and therapeutic sensitivity in pancreatic cancer.

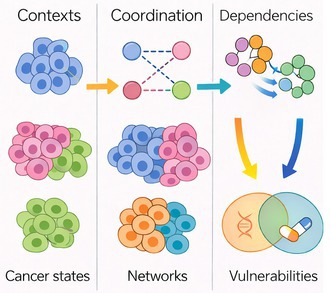

**Abbreviations**
DEG: differentially expressed gene
FDR: false discovery rate
HR: hazard ratio
IGE: integrated growth‐energy module
IL2: IL‐2–linked immune evasion module
KM: Kaplan–Meier
MPC: multi‐pathway collective invasion module
OS: overall survival
PDAC: pancreatic ductal adenocarcinoma
PRISM: profiling relative inhibition simultaneously in mixtures
SAT: stress‐adaptive transcription module
scRNA‐seq: single‐cell RNA sequencing
UMAP: uniform manifold approximation and projection

## 1 Introduction

Dynamic reorganization of gene regulatory networks gives rise to diverse transcriptional programs that underlie cellular heterogeneity in cancer [1, 2, 3, 4]. These network‐level processes enable tumor cells to adopt distinct transcriptional configurations associated with proliferation, differentiation, invasion, or therapeutic resistance [2, 3, 4, 5, 6]. In pancreatic ductal adenocarcinoma (PDAC), such regulatory complexity contributes to its extensive heterogeneity and treatment resistance, yet the structure and functional relevance of these adaptive programs remain poorly understood [7, 8, 9, 10]. PDAC tumors encompass a continuum of transcriptional states—from classical to basal‐like—and can shift between them under selective pressures, reflecting profound regulatory plasticity [11, 12]. These transcriptional state transitions are tightly coupled to metabolic reprogramming, as PDAC cells adapt their utilization of glucose, glutamine, lipids, and mitochondrial pathways to survive within a hypovascularized, nutrient‐limited microenvironment [13, 14, 15]. However, the specific patterns of gene–gene coordination that define these adaptive states, and how they correspond to experimentally defined dependencies or therapeutic vulnerabilities, have not been systematically mapped.

Although individual adaptive processes such as epithelial–mesenchymal transition (EMT), lineage switching, and immune evasion have been well described [16, 17, 18], most were discovered through targeted biological hypotheses rather than unbiased network‐level analyses. Conventional single‐cell analyses, including clustering and differential expression, identify stable transcriptional states but do not capture how regulatory relationships between genes change as cells transition between them [19, 20]. Similarly, static co‐expression networks describe average connectivity but fail to reveal the dynamic rewiring of gene–gene coordination that underlies transcriptional adaptation [21]. As a result, emergent and previously unrecognized regulatory programs—those not aligned with predefined biological categories—may remain undetected.

To address these limitations, we developed a gene–gene co‐expression‐divergence framework that quantifies shifts in regulatory coordination between malignant subpopulations in single‐cell RNA‐seq data. This approach measures how the correlation between each gene pair varies across cellular contexts, revealing dynamic changes in co‐expression that define adaptive transcriptional modules without reliance on prior pathway knowledge [22].

We applied this framework to a dataset of 77 155 malignant cells from 42 PDAC tumors spanning untreated primaries, post‐chemotherapy samples, and metastases. The analysis revealed four distinctive adaptive modules involving IL2‐linked immune evasion, stress‐adaptive transcription, multi‐pathway collective invasion, and integrated growth‐energy regulation. To assess the functional and translational relevance of these modules, we cross‐referenced them with CRISPR‐based gene dependency and drug‐sensitivity data from DepMap [23, 24, 25] and evaluated their transcriptional counterparts in independent clinical PDAC cohorts. Together, these analyses delineate the network‐level logic by which PDAC cells create adaptive transcriptional programs, linking single‐cell regulatory rewiring to potential therapeutic vulnerabilities.

## 2 Materials and methods

### 2.1 Data sources and preprocessing

Single‐cell RNA‐seq data were obtained from two published pancreatic ductal adenocarcinoma (PDAC) datasets: Peng et al. (CRA001160) and Werba et al. (GSM6204109) [12, 26]. Clinical metadata were compiled for all PDAC samples included in the analysis, including age at diagnosis, sex, histologic differentiation, tumor location, specimen type, TNM classification, stage, and available pathological features. Two independent scRNA‐seq cohorts were analyzed which differed in study design and case ascertainment. The Werba et al. cohort included both primary pancreatic tumors and metastatic liver samples, with a substantial fraction of advanced‐stage cases and biopsy‐derived specimens. In contrast, the Peng et al. cohort consisted exclusively of surgically resected primary pancreatic tumors and was enriched for localized disease (predominantly stages IB–IIB), with comprehensive pathological annotation including perineural invasion, vascular invasion, and peripancreatic infiltration. A summary comparison of clinical characteristics across cohorts is provided in Table S1.

For the Peng dataset, raw fastq files were downloaded from the Genome Sequence Archive. For the Werba dataset, BAM files were retrieved from GEO and converted to fastq using bamtofastq (10× Genomics). The majority of malignant cells analyzed were derived from treatment‐naive primary pancreatic tumors (29 out of 42). Only four samples across the integrated scRNA‐seq cohorts were obtained following systemic therapy, and a minority of samples were derived from liver metastases (*n* = 9), with the remainder originating from primary pancreatic tumors. Because post‐treatment and metastatic samples were limited in number, they were not analyzed as separate strata. All fastq files were processed with Cell Ranger (10× Genomics, v7.2), aligned to the GRCh38 human reference genome, and aggregated at the sample level. Ambient RNA contamination was removed using cellbender remove background [27], testing nominal false‐positive rates (0.01, 0.05, 0.1), and selecting optimal fits following developer recommendations. Doublets were identified using scrublet [28]. Downstream filtering and preprocessing were performed with scanpy [29], removing cells with low UMI counts, extreme gene counts, or high mitochondrial content, followed by log‐normalization. Cells were retained if they expressed at least 200 genes, fewer than 8000 genes, and less than 10% mitochondrial transcripts. Genes expressed in fewer than three cells were excluded from downstream analyses.

### 2.2 Identification of malignant cells

To identify malignant epithelial cells, we performed Leiden clustering on the cleaned dataset and annotated cell types using canonical lineage markers. Copy number variation profiles were inferred using infercnvpy [30], using stromal and immune populations as normal references. Clusters exhibiting broad chromosomal amplifications or deletions were classified as malignant ductal cells. This procedure yielded 77 155 malignant cells across 42 tumors, used for all subsequent analyses.

### 2.3 Integration, clustering, and imputation

To integrate malignant cells across patients, we applied Harmony batch correction on the PCA embedding [31]. Malignant tumor cells from all samples were concatenated into a single AnnData object using sc.concat with an outer gene union, and both sample identity and study of origin were stored as metadata. Genes detected in fewer than five cells were removed prior to dimensionality reduction. Principal component analysis (PCA) was performed on the concatenated, unintegrated data, followed by batch correction using Harmony in PCA space with sample identity as the batch variable. The Harmony‐corrected PCA embedding (X_pca_harmony) was used for all downstream analyses, including neighborhood graph construction, UMAP visualization, and Leiden clustering.

Batch‐mixing effectiveness was assessed quantitatively in PCA space, the space in which Harmony operates, using silhouette scores computed with respect to batch labels. After Harmony integration, silhouette scores were low for both sample identity (−0.15) and study of origin (−0.01), consistent with effective reduction of batch‐associated structure. For figure presentation, batch mixing before and after integration is visualized using UMAP embeddings constructed from Harmony‐corrected PCs (Fig. S1). UMAP visualizations were used because they more clearly illustrate changes in sample‐ and study‐specific structure following integration, while quantitative assessment was performed in PCA space.

To evaluate preservation of biological signal, established PDAC subtype signatures and functional program scores were projected onto the postintegration embedding. These scores retained smooth, heterogeneous gradients across the malignant cell manifold rather than collapsing into homogeneous clusters (Fig. S2), indicating that Harmony integration reduced technical batch effects without erasing biologically meaningful transcriptional variation.

Leiden clustering of the integrated dataset produced 28 malignant clusters (Table S2), with the 10 largest representing approximately 70% of all malignant cells. Imputation of missing values was performed using ALRA, which preserves true zero counts while correcting dropout [32]. Imputation was restricted to the 10 dominant clusters to maintain consistent statistical power for downstream correlation and divergent‐edge analyses.

### 2.4 Divergent‐edge network construction

To characterize state‐dependent gene–gene coordination, we constructed cluster‐specific correlation matrices and identified gene pairs whose correlation structure varied across malignant cell states. For each Leiden cluster c, we computed a gene–gene Pearson correlation matrix $R^{\left(c\right)}$ using expression values across cells assigned to that cluster. This resulted, for each gene pair $\left(i,j\right)$, in a vector of correlation coefficients across clusters,

$$r_{\mathrm{ij}}=\left(r_{\mathrm{ij}}^{\left(1\right)},r_{\mathrm{ij}}^{\left(2\right)},\ldots ,r_{\mathrm{ij}}^{\left(C\right)}\right),$$

where C denotes the number of clusters analyzed.

We define a divergent edge as a gene pair whose correlation coefficients differ substantially across clusters, indicating context‐dependent coordination. Divergence was quantified using the range of correlation values across clusters,

$$D_{\mathrm{ij}}=\max\left(r_{\mathrm{ij}}\right)-\min\left(r_{\mathrm{ij}}\right).$$

Gene pairs were ranked by $D_{\mathrm{ij}}$, and those with the highest divergence scores were retained as divergent edges for downstream network construction and visualization. We used the range as a simple, nonparametric measure of divergence because it directly captures the maximal contrast in gene–gene coordination across cell states and is well suited to ranking gene pairs when the number of clusters is modest. The input to the divergent‐edge algorithm is a set of cluster‐specific gene–gene correlation matrices, and the output is a ranked list of gene pairs prioritized by cross‐cluster divergence. This framework is intended to support comparative analysis of transcriptional coordination across cellular contexts and does not assume a specific mechanistic model of gene regulation.

Below is the divergent‐edge algorithm (pseudocode):

Input: normalized expression matrix 𝑋 (cells × genes); malignant cell cluster labels *c* ∈

{1, …, *C*} (e.g., Leiden clusters)

Output: ranked list of divergent gene pairs (edges) with divergence scores 𝐷_!″_

1. Compute cluster‐specific correlation matrices
  For each cluster *c* = 1, …, *C*:
2. Subset cells: *X* ^(c)^ ← *X*[cells with label *c*]
3. Compute Pearson correlations across cells for all gene pairs: *R* ^(c)^ ← corr(*X* ^(c)^)
4. Score divergence for each gene pair
  For each gene pair (*i*, *j*) with *i* < *j*:
5. Collect correlations across clusters: **r**_*ij*_ ← {*R*_*ij*_^(1)^, *R*_*ij*_^(2)^, …, *R*_*ij*_^(C)^}
6. Compute divergence (range across clusters):

$${\text{𝐷}}_{\mathrm{ij}}\leftarrow \max\;\left(r_{\mathrm{ij}}\right)-\min\;\left(r_{\mathrm{ij}}\right)$$

1. Rank and select divergent edges
2. Rank all gene pairs by 𝐷_*ij*_ in descending order
3. Retain the top‐ranked pairs (or those above a chosen cutoff) as the divergent edges for downstream network analyses

Notes: The procedure yields a state‐conditioned gene–gene coordination network in which edges represent gene pairs whose correlation structure differs most across clusters.

Cluster‐specific Pearson correlation matrices were computed for each of the 10 largest malignant clusters. Gene pairs were scored for divergence across clusters using the range of correlation values, and high‐divergence edges were aggregated to construct the network shown.

Divergent‐edge networks were constructed by retaining edges ranked by absolute divergence score. To assess robustness to threshold choice, analyses were repeated using three representative thresholds spanning two orders of magnitude: the top 2000, 20 000, and 200 000 divergent edges. These values were selected to capture sparse, intermediate, and dense network regimes while remaining computationally tractable. Across this range of thresholds, the major transcriptional modules identified by the divergent‐edge framework were consistently recovered, with similar gene composition and functional enrichment. Threshold choice primarily affected network density and visualization complexity rather than the identity of core modules. Because the primary objective of the analysis was to identify robust, recurrent coordination modules rather than to optimize graph‐theoretic properties, formal modularity scores and network statistics were not used as selection criteria. Network topology was therefore evaluated qualitatively with respect to module stability across thresholds rather than quantitatively optimized for a single metric.

Within each module, genes were classified as core if they exhibited strong‐positive correlations with a large fraction of other module genes, forming a densely interconnected subnetwork. Genes were classified as peripheral if they showed weak, negative, or context‐specific correlations with core genes and did not participate in the dominant positively correlated block. This classification reflects relative network position and coordination structure rather than absolute expression level. The term ‘regulatory feedback architecture’ refers to the presence of tightly coordinated gene sets characterized by mutual‐positive correlations, consistent with collective regulation or shared upstream control, together with peripheral genes that interact asymmetrically or antagonistically with this core. The analysis does not infer causal directionality but instead captures patterns of coordinated or opposing gene–gene behavior across contexts.

### 2.5 DepMap dependency analysis

RNA‐seq expression profiles from pancreatic and gastrointestinal cancer cell lines in DepMap 24Q4 [24, 25] were used to compute module activity scores by ssGSEA (gseapy). Module scores were correlated (Spearman) with Chronos CRISPR–Cas9 dependency scores for ~ 17 000 genes. For dependency and drug‐response analyses, we used a screening threshold of |ρ| > 0.3 and FDR < 0.1 to support hypothesis generation. More stringent thresholds yielded few associations, consistent with limited statistical power across PDAC cell lines and the imperfect correspondence between tumor‐derived transcriptional programs and functional assays performed *in vitro*; accordingly, these correlations are interpreted as candidate vulnerabilities for prioritization rather than definitive dependencies.

### 2.6 PRISM drug‐response analysis

Drug‐sensitivity data (AUC values) from the PRISM Repurposing Primary Screen were correlated with module activity across PDAC lines using Spearman correlation. Compounds with ρ < −0.3 and FDR < 0.1 were designated as candidate selective sensitivities. Drugs were grouped by mechanism of action to identify pathway‐level response patterns.

### 2.7 Clinical cohorts and survival analysis

Bulk RNA‐seq data from the TCGA‐PAAD cohorts were normalized and scored using single‐sample gene set enrichment analysis (ssGSEA) with the four module gene sets. For TCGA‐PAAD, Kaplan–Meier survival curves were generated using *z*‐scored module activity values and optimal cut points selected by maximizing the log‐rank statistic (maxstat), subject to a minimum group proportion constraint. *P*‐values for cut point‐based Kaplan–Meier analyses were computed using the maxstat procedure to account for multiple testing across candidate thresholds. These analyses were used to illustrate relative survival stratification across modules and module combinations. Multivariable survival associations were assessed using Cox proportional hazards models implemented in the R survival package, incorporating continuous module activity scores and adjusting for clinicopathologic covariates including AJCC pathologic stage and tumor grade. Module activity differences between classical and basal‐like tumor subtypes were evaluated using the Wilcoxon rank‐sum test.

### 2.8 Software and reproducibility

All analyses were performed using python 3.10 and r 4.3 with standard scientific libraries (Scanpy, NumPy, Pandas, Harmony‐Python, NetworkX, igraph, gseapy). Figures were generated using matplotlib and seaborn. All code is available upon request.

## 3 Results

### 3.1 Mapping dynamic co‐expression modules in PDAC

To identify transcriptional programs that vary across malignant cell states, we analyzed 77 155 single PDAC cells from 42 tumors spanning untreated primary lesions, postchemotherapy samples, and liver metastases. Following integration with harmony [31] and imputation with alra [32] to preserve biological zeros, Leiden clustering of malignant cells yielded 28 clusters with a strongly right‐skewed size distribution (Fig. S3). The 10 largest clusters together comprised approximately 69% of malignant cells, while the remaining clusters formed a long tail of progressively smaller populations (Table S2), consistent with heavy‐tailed cluster size distributions commonly observed in large single‐cell datasets. Given the limited cell numbers in smaller clusters and the reliance of correlation‐based analyses on adequate sample size for stability, downstream divergent‐edge analysis was restricted to the 10 largest clusters, which provided sufficient numbers of cells for robust estimation of gene–gene correlations.

**Fig. 1:**
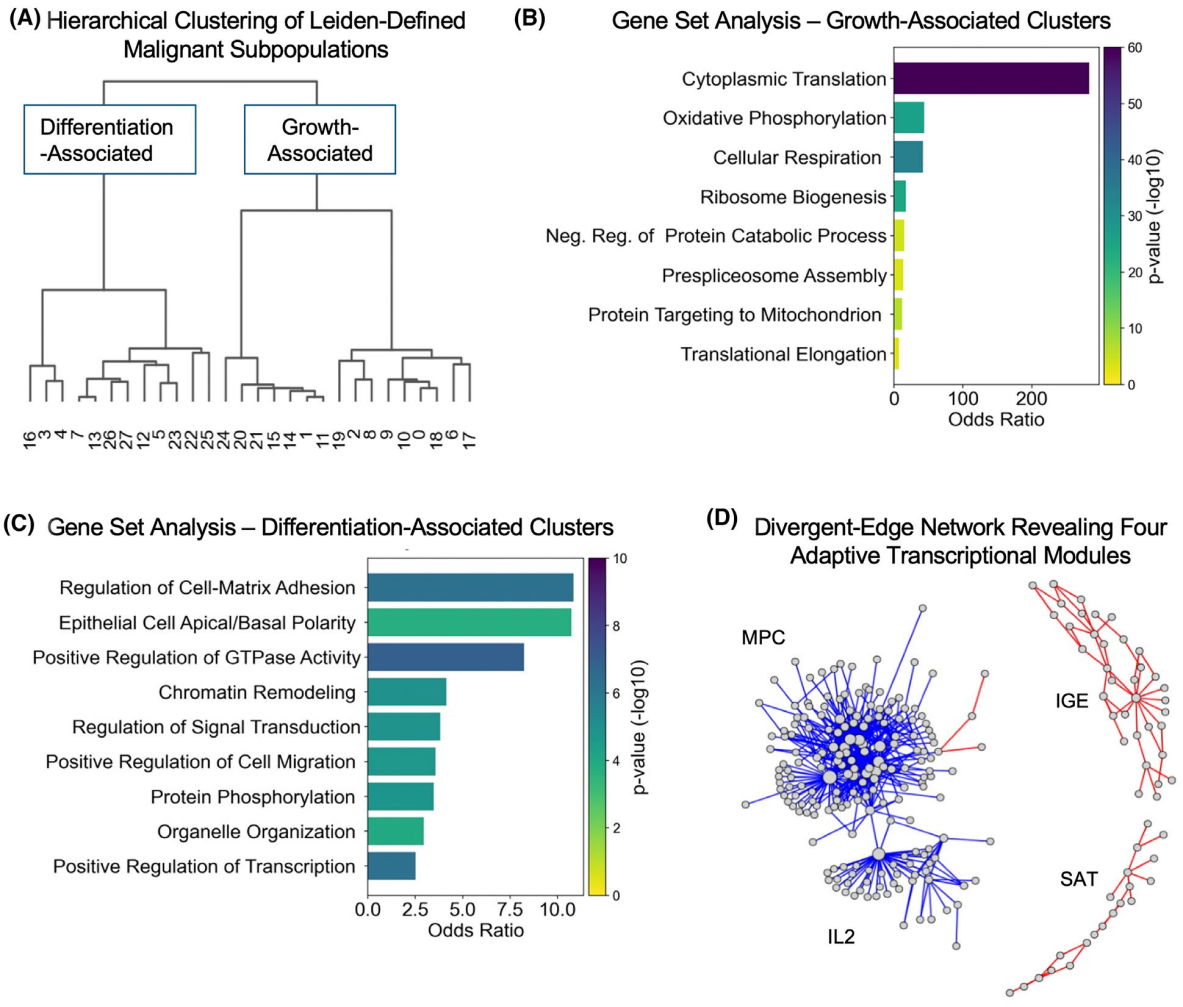
Growth‐differentiation structure and divergent‐edge network architecture in PDAC. (A) Hierarchical clustering of Leiden‐defined malignant subpopulations following Harmony integration and ALRA imputation, separating transcriptionally differentiation‐associated clusters (left; 32%, 28 968 cells) from growth‐associated clusters (right; 62%, 48 187 cells). (B) Gene Ontology Biological Process (GO‐BP) over‐representation analysis for the growth‐associated transcriptomes. (C) GO‐BP over‐representation analysis for the differentiation‐associated transcriptomes. (D) The divergent‐edge network constructed from gene pairs whose correlation coefficients vary across the 10 largest malignant clusters, aggregated to highlight recurrent patterns of context‐dependent gene–gene coordination. There are four adaptive modules: integrated growth‐energy (IGE), stress‐adaptive transcription (SAT), IL‐2–linked immune evasion (IL2), and multi‐pathway collective invasion (MPC). The edges arising from the most prominent Leiden cluster pairing are shown in blue, and those from the second‐most prominent pairing are shown in red.

Hierarchical partitioning separated clusters into two principal groups (Fig. 1A). One group was enriched for growth‐related processes—including protein synthesis, oxidative phosphorylation, and mRNA splicing—while the other showed enrichment for differentiation‐associated pathways such as signal transduction, cell–matrix interactions, and chromatin remodeling (Fig. 1B,C). For clarity, subgroup labels are intended as descriptive summaries of enriched functional themes rather than definitive biological state assignments. Figure 1A–C define the malignant cell‐state structure used to stratify cells into contexts for downstream divergent‐edge analysis, rather than serving as direct inputs to a cluster‐specific network.

We next examined how gene–gene coordination differs across subpopulations. For the 10 largest clusters (representing ~ 69% of malignant cells), we computed pairwise Pearson correlations between genes and quantified their co‐expression divergence. This metric captures context‐specific regulatory shifts—reflecting the regulatory rewiring that underlies cancer cell plasticity [1, 2, 3, 4, 16, 17]. The 10 clusters selected for cluster‐specific gene–gene correlation analysis contained a median of 5286 cells per cluster (range: 7274–3500), providing sufficient sample sizes for stable estimation of correlations.

Using the most divergent gene pairs, we constructed transcriptional divergence networks. Across a wide range of thresholds, community detection consistently identified four recurrent co‐expression modules: an integrated growth‐energy (IGE) program, a stress‐adaptive transcription (SAT) program, an IL‐2–linked immune‐evasion (IL2) program, and a multi‐pathway collective invasion (MPC) program (Fig. 1D). These mirror transcriptional states described across spatial and single‐cell PDAC atlases [5, 6, 7, 8, 9, 10, 11, 12].

These findings reveal that PDAC heterogeneity is structured around a limited number of dynamic transcriptional modules, each reflecting coordinated rewiring of gene co‐expression across malignant cell states. As shown below, integrating these modules with functional genomic datasets reveals that they organize into two orthogonal adaptive axes with distinct biological and clinical implications.

### 3.2 Integrated growth‐energy module

**Fig. 2:**
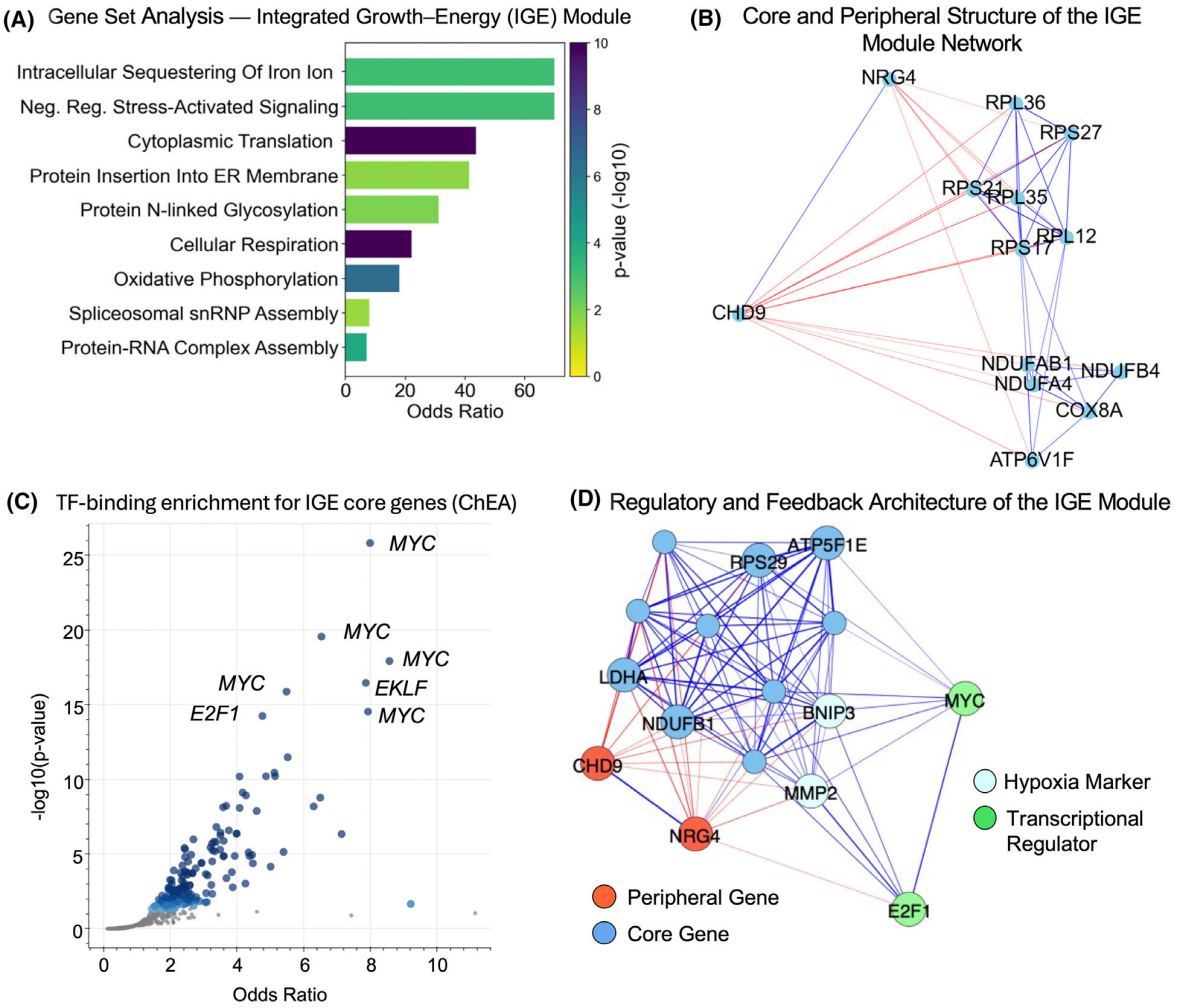
Characterization of the integrated growth‐energy (IGE) module. (A) Gene Ontology Biological Process (GO‐BP) over‐representation analysis of IGE core genes, highlighting enrichment for translation, oxidative phosphorylation, cellular respiration, and ER‐associated protein processing. (B) Subnetwork illustrating the relationship between core biosynthetic genes and peripheral regulators *CHD9* and *NRG4*. Blue edges denote positive correlations and red edges denote negative correlations; edge width reflects correlation magnitude. (C) Transcription factor enrichment analysis (ChEA), identifying *MYC*, *E2F1*, and related regulators as the top predicted upstream drivers of the IGE program. (D) Integrated regulatory subnetwork showing coordinated expression of core IGE genes with *MYC* and *E2F1* (green), co‐expression of hypoxia‐associated genes (*BNIP3, MMP2*; light cyan), and opposing correlations with peripheral negative regulators *CHD9* and *NRG4* (red).

The integrated growth‐energy (IGE) module represents a transcriptional shift toward enhanced biosynthetic and metabolic activity within a defined subset of malignant PDAC cells. Core genes in this module include numerous ribosomal proteins (20%) and mitochondrial oxidative phosphorylation components (12%) (Table S3). Gene Ontology enrichment analyses highlighted translation, cellular respiration, endoplasmic reticulum protein processing, and mRNA splicing (Fig. 2A), consistent with the biosynthetic programs associated with proliferative PDAC states described previously [5, 7].

The module was identified within the 2000‐divergent‐edge network and comprises 118 core genes and 26 peripheral genes. Expression patterns across Leiden clusters revealed that the module is initiated within differentiation cluster 5, where a subset of cells displays strong, coordinated activation of mitochondrial and ribosomal genes. This subpopulation transitions toward transcriptional profiles characteristic of growth cluster 9, suggesting that the IGE module captures an adaptive shift from a more differentiated epithelial state to a biosynthetically active, anabolic phenotype.

Peripheral regulators displayed distinct inverse relationships with the core program. In particular, *CHD9*, a chromatin remodeler, and *NRG4*, a growth factor implicated in epithelial signaling balance, were negatively correlated with core gene expression (Fig. 2B). Their inverse association suggests a built‐in negative‐feedback circuit that may modulate excessive biosynthetic activation.

Transcription‐factor motif enrichment using ChEA identified *MYC* and *E2F1* as the most prominent upstream regulators (Fig. 2C), consistent with their central roles in driving ribosome biogenesis, mitochondrial function, and cell‐cycle progression in PDAC [5, 7]. Two canonical MYC targets—*CDK4* and *LDHA*—were tightly correlated with the IGE core gene set (Fig. 2D), reinforcing the linkage between the module and *MYC*–*E2F*‐driven anabolic growth.

The co‐expression of *BNIP3* and *MMP2*, markers of hypoxia and ECM remodeling, suggests that localized oxygen limitation may promote activation of this biosynthetic program. This pattern aligns with reports that hypoxia stabilizes *MYC*‐linked metabolic rewiring in PDAC, fostering reliance on oxidative phosphorylation and ribosomal output under nutrient stress.

Collectively, the IGE module delineates a coordinated transcriptional transition characterized by elevated mitochondrial function, ribosome biogenesis, and *MYC*–*E2F*–mediated anabolic signaling. The presence of counter‐regulatory elements such as *CHD9* and *NRG4* indicates that this growth‐energy program is not simply a unidirectional proliferative state but rather an actively regulated, feedback‐constrained adaptive phenotype within PDAC.

### 3.3 Stress‐adaptive transcriptional module

The stress‐adaptive transcription (SAT) module defines a coordinated gene expression program activated in a discrete subset of PDAC cells undergoing metabolic, proteotoxic, or environmental stress. Identified in the 20 000‐divergent‐edgenetwork, the module consists of 185 core and 25 peripheral genes, showing the highest correlation within differentiation cluster 5. Expression was confined to a small subpopulation, indicating that SAT activation marks a specialized, stress‐resilient malignant niche, rather than a global feature of the tumor.

**Fig. 3:**
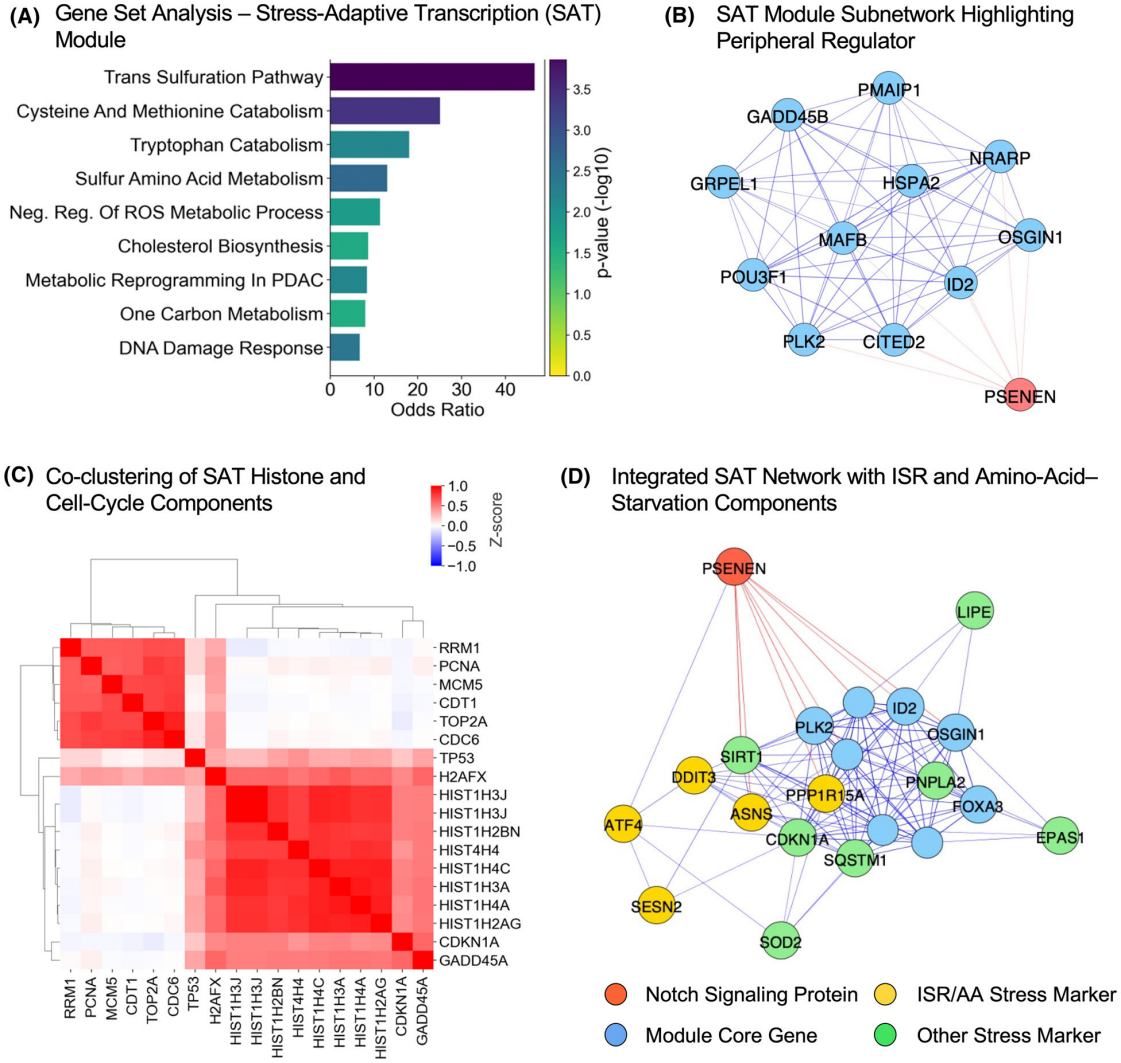
Characterization of the stress‐adaptive transcription (SAT) module. (A) GO‐BP over‐representation analysis of SAT core genes, highlighting metabolic stress, trans‐sulfuration, amino‐acid catabolism, and redox‐associated pathways. (B) SAT subnetwork showing positively correlated core genes (blue, *r* > 0.3) and the negatively correlated peripheral regulator *PSENEN* (red). Edge colors reflect correlation sign. (C) Correlation heatmap illustrating strong co‐expression (*r* > 0.3) between SAT core genes and DNA damage/repair genes, with minimal correlation (*r* < 0.05) to DNA‐replication genes. (D) Integrated SAT subnetwork showing coordinated expression (*r* > 0.3) of core genes with markers of the integrated stress response (ISR; yellow), amino‐acid–starvation stress (green), and other stress contexts such as hypoxia and autophagy (cyan), alongside negatively correlated peripheral nodes (red).

Gene set enrichment initially mapped the module to broad metabolic and stress‐related processes (Fig. 3A), but closer inspection revealed a cohesive signature of the integrated stress response (ISR) and amino‐acid deprivation, two pathways strongly implicated in PDAC survival under nutrient and hypoxic pressure [6, 11]. The module includes numerous effectors of oxidative defense, metabolic rewiring, and DNA damage response (Table S3). A strongly co‐expressed subnetwork (Fig. 3B) contained canonical ISR mediators—*ATF4, DDIT3* (*CHOP*), *PPP1R15A*, and *SESN2*—consistent with PERK–eIF2α–ATF4 signaling that enables PDAC cells to tolerate ER stress and disrupted protein homeostasis [17].

A notable feature of the SAT module is its regulatory heterogeneity: ~ 24% of core genes encode long noncoding RNAs, and an additional set of 14 histone genes correlate more strongly with DNA damage response genes than with replication markers (Fig. 3C). This pattern implicates chromatin‐based stress regulation, rather than classical S‐phase‐linked histone synthesis, as a driver of this transcriptional state.

Correlation analysis with canonical stress pathways further refined the module's selectivity: strong associations were observed with amino‐acid starvation, autophagy, hypoxia, and lipid deprivation, but not with glucose starvation (*r* < 0.2) (Fig. 3D). Among peripheral genes, *PSENEN*, a Notch‐signaling component, was inversely correlated with core SAT genes, suggesting a feedback mechanism in which Notch signaling may restrain prolonged ISR activation—a relationship consistent with reports that Notch–stress crosstalk shapes PDAC cell‐state transitions [7].

Together, these findings define SAT as a unified stress‐adaptation circuit that equips a minority of PDAC cells to withstand metabolic deprivation, ER stress, and proteotoxic load. Its restriction to a small cellular niche highlights an important but previously underappreciated axis of transcriptional heterogeneity in PDAC—one that may contribute disproportionately to resistance under therapeutic pressure.

### 3.4 IL2‐linked immune evasion module

The IL2‐linked immune‐evasion (IL2) module represents a distinct cytokine‐associated program that coordinates the expression of multiple immune‐modulatory genes within malignant PDAC cells. Identified within the 2000‐divergent‐edge network, the module comprises 106 core and 23 peripheral genes, with core expression restricted to a small subpopulation in growth‐associated cluster 1—a striking contrast to the stress‐linked location of the SAT module.

**Fig. 4:**
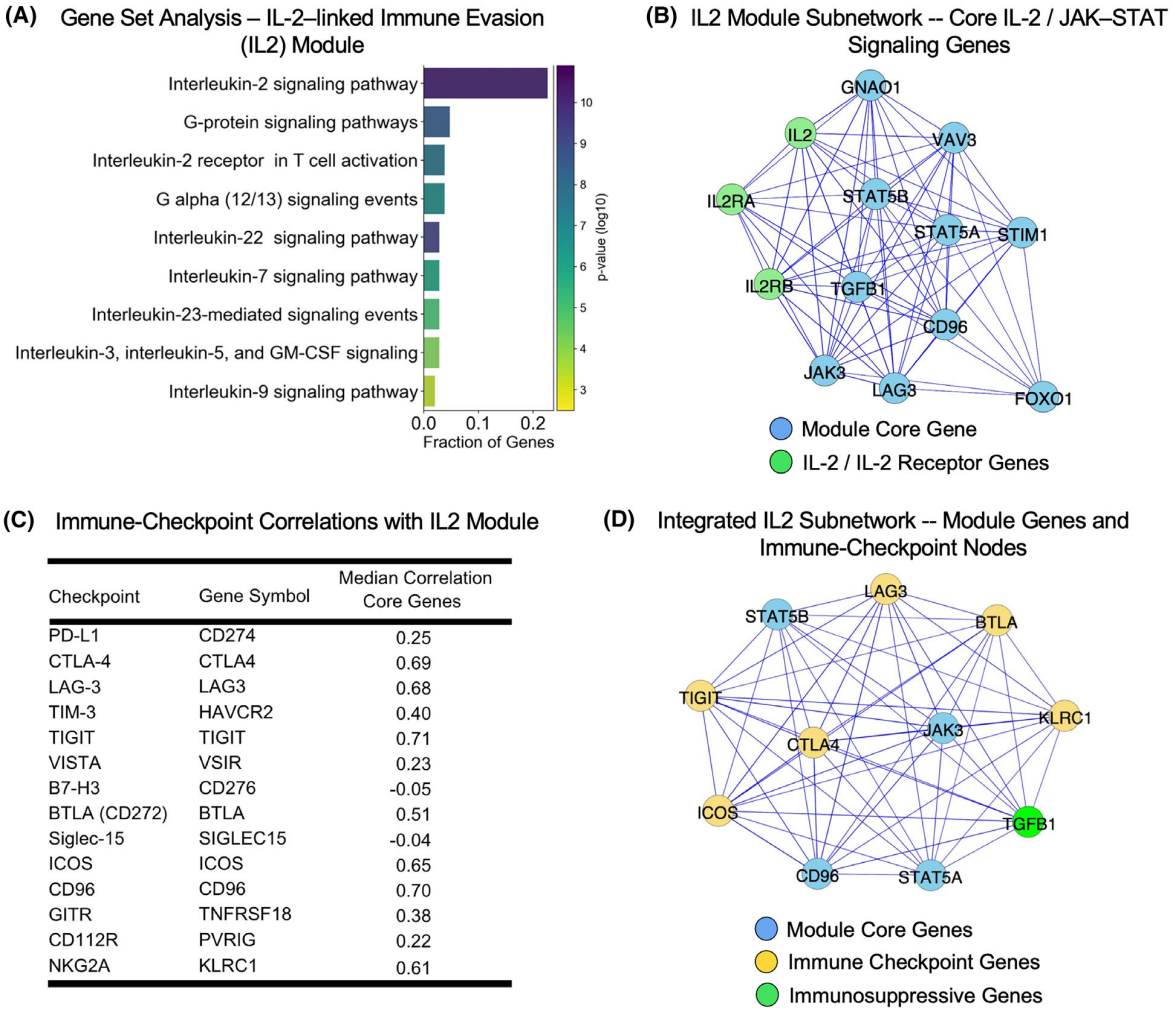
Characterization of the IL‐2–linked immune evasion (IL2) module. (A) GO‐BP over‐representation analysis of core IL2‐module genes, identifying enrichment for IL‐2, G‐protein, and T‐cell‐associated cytokine signaling pathways. (B) Subnetwork of IL2‐module core genes (*r* > 0.3), highlighting coordinated expression of IL‐2 receptor subunits (*IL2RA, IL2RB*), downstream mediators (*JAK3, STAT5A/B*), and signaling adaptors. (C) Correlation table summarizing checkpoint molecules and their median correlations with IL2‐module core genes, revealing coordinated expression with multiple inhibitory and co‐stimulatory receptors (e.g., *CTLA4, LAG3, TIGIT, KLRC1*). (D) Integrated subnetwork showing correlated expression (*r* > 0.6) between IL2 core genes (blue), immune checkpoint genes (yellow), and immunosuppressive genes (green), illustrating the module's linkage to a coordinated immune evasion program.

Although initial GO analysis categorized the module under general immune signaling, deeper pathway analysis using NCATS BioPlanet revealed a selective enrichment for the interleukin‐2 (IL‐2) signaling pathway, rather than other IL‐family signals (Fig. 4A). This was further supported by coordinated expression of *IL2*, *IL2RA*, *IL2RB*, and the downstream transcription factors *JAK3*, *STAT5A*, and *STAT5B* (Fig. 4B). Together, these associations delineate a noncanonical *IL‐2/STAT5* axis within tumor cells, distinct from the more commonly described *IFN‐γ/JAK1/2* or *IL‐6/STAT3* pathways in PDAC immune evasion [9, 10].

Based on the observation that the core gene *CD96* is an immune checkpoint molecule [33], we examined whether the expression of other immune checkpoint molecules correlated with the expression of core module genes. Among the 13 additional immune checkpoint genes analyzed, six showed strong positive correlation (*r* > 0.6) with core genes, two demonstrated moderate correlation (*r* > 0.4), and four additional genes showed weak but statistically significant correlation (*r* > 0.2) (Fig. 4C). These findings suggest that this gene module contributes to immune evasion by promoting the expression of multiple immune checkpoint molecules, thereby enabling tumor cells to escape immune destruction.

The subnetwork of the strongly correlated immune checkpoint genes along with select core module genes is shown in Fig. 4D. Additionally, the immunosuppressive cytokine *TGFB1* was also strongly correlated with core module gene expression, placing the module's role in a broader immune‐modulatory phenotype (Fig. 4D).

### 3.5 Multi‐pathway collective invasion module

The multi‐pathway collective invasion (MPC) module captures a transcriptional program underlying coordinated, junction‐retaining modes of PDAC invasion. Identified within the 2000‐divergent‐edge network, it is the largest of the four modules, comprising 304 core and 15 peripheral genes, with expression in 30–40% of cells within the initiating cluster. This broad activation contrasts with the niche‐restricted SAT and IL2 programs and suggests that collective invasion is a dominant, population‐level strategy in many PDAC tumors.

**Fig. 5:**
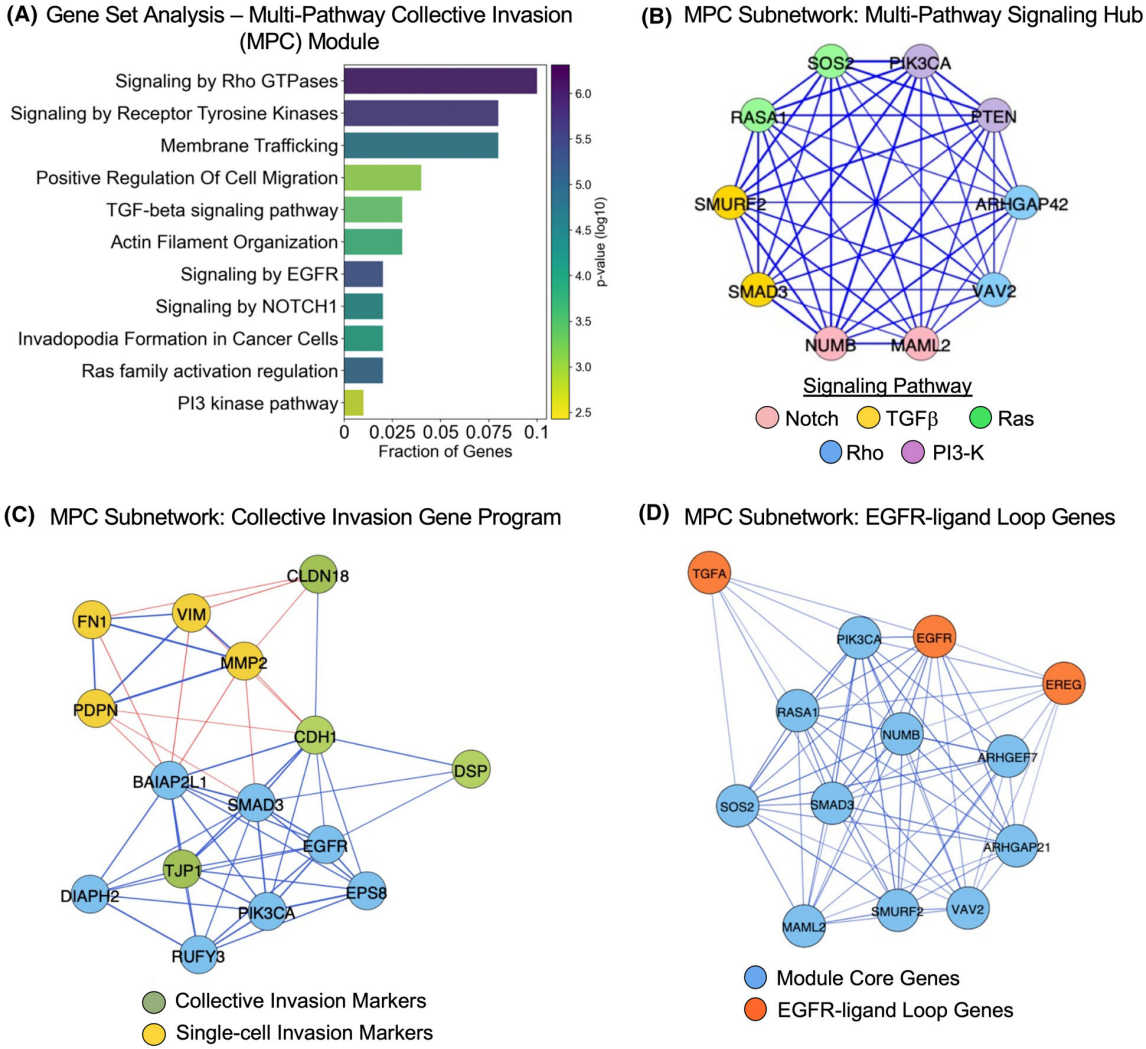
Characterization of the multi‐pathway collective invasion (MPC) module. (A) GO‐BP analysis of MPC core genes shows enrichment for Rho‐GTPase signaling, receptor tyrosine kinase pathways, membrane trafficking, TGF‐β and EGFR signaling, and actin filament organization—processes supporting epithelial‐retaining, coordinated invasion. (B) MPC (sub)network highlighting a densely interconnected signaling hub linking RTK (*EGFR/PIK3CA*), Rho‐GTPase (*ARHGEF/DIAPH*), TGF‐β (*SMAD2/3*), and Notch (*NUMB/MAML2*) components; blue/red edges denote positive/negative co‐expression. (C) Expanded MPC (sub)network illustrating the collective invasion gene program, where epithelial markers (*CDH1, TJP1, CLDN18, DSP*) form a correlated module inversely related to mesenchymal markers (*VIM, FN1*, *MMP2, PDPN*). (D) MPC (sub)network showing autocrine *EGFR–EREG* and *EGFR–TGFA* pairs, indicating a self‐reinforcing EGFR signaling loop integrated with core MPC pathways.

Gene set enrichment analyses linked MPC to actin filament organization, membrane trafficking, cell migration, focal adhesion, and invadopodium formation—hallmarks of PDAC collective invasion [8, 10] (Fig. 5A). The module spans multiple signaling pathways, including Rho GTPase, Ras, receptor tyrosine kinases (RTKs), PI3K, TGF‐β, and Notch, with both activators and inhibitors showing coordinated positive correlations (Fig. 5B). This broad coregulation suggests that PDAC collective invasion is sustained not by single‐pathway activation but by a finely tuned, multi‐pathway signaling equilibrium.

To distinguish collective from single‐cell modes of invasion, we examined correlations with established markers from both categories. MPC activity correlated strongly with markers of collective migration—*CDH1, DSP, TJP1, CLDN18* (*r* > 0.4)—and was inversely associated with single‐cell/mesenchymal markers such as *VIM, MMP2, FN1*, and *PDPN* (Fig. 5C). These relationships indicate that MPC does not correspond to EMT‐driven dissemination but rather supports a junction‐retaining, leader–follower mode of tumor invasion that preserves epithelial architectures during migration.

The identification of autocrine EGFR–EREG and EGFR–TGFA ligand–receptor pairs (Fig. 5D) provides mechanistic support for self‐reinforcing EGFR signaling loops that sustain directional, collective invasion, consistent with prior observations in PDAC [6]. Network topology confirmed an integrated architecture (modularity = 0.09), with cytoskeletal and signaling genes interwoven across subclusters (Fig. S4A). Membrane‐trafficking regulators—including RAB family GTPases, GDI1/2, and MYO5B—were strongly connected to RTK and Rho nodes, indicating that vesicle transport and membrane dynamics are central to maintaining invasive polarity (Fig. S4B).

Together, the MPC module defines a broad, multi‐pathway invasion program in PDAC, characterized by synchronized cytoskeletal remodeling, RTK/Rho/TGF‐β signaling, and membrane‐trafficking dynamics. Its high prevalence suggests that collective invasion represents a primary invasive strategy in PDAC, with implications for metastasis, drug resistance, and stromal engagement.

### 3.6 Relationship between divergent‐edge modules, PDAC subtype signatures, and functional programs

**Fig. 6:**
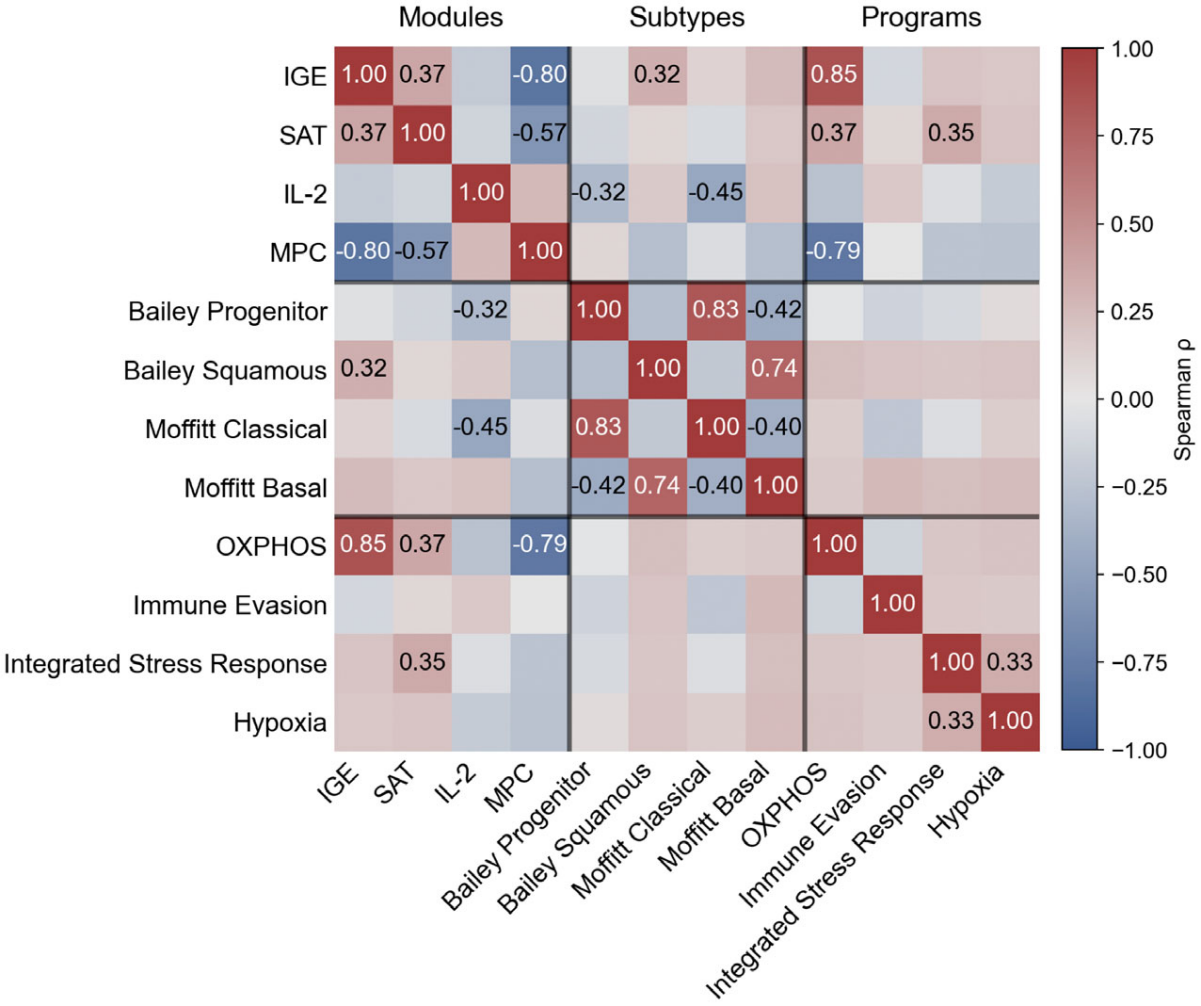
Relationship between divergent‐edge modules, PDAC subtype signatures, and functional programs. Heatmap showing pairwise Spearman correlations between divergent‐edge module scores (IGE, SAT, IL‐2, MPC), established PDAC transcriptional subtype signatures (Bailey progenitor, Bailey squamous, Moffitt classical, Moffitt basal), and functional gene expression programs (OXPHOS, immune evasion, integrated stress response, hypoxia). Correlations were computed across malignant cells using module and program scores derived from single‐cell transcriptomic data. Positive correlations are shown in red and negative correlations in blue, with color intensity reflecting correlation magnitude. Values are annotated for selected comparisons to highlight key relationships. Vertical and horizontal lines delineate module, subtype, and program groupings. This analysis demonstrates that divergent‐edge modules align only partially with established PDAC subtypes and instead reveal higher‐order functional relationships, including a strong association between the biosynthetic–metabolic (IGE) module and OXPHOS programs, and an inverse relationship between metabolic and stress‐adaptive or invasive programs.

To clarify how the divergent‐edge modules relate to previously described PDAC molecular subtypes and canonical biological programs, we computed Spearman correlations between single‐cell module scores and representative PDAC subtype signature scores, as well as established functional program scores (Fig. 6). PDAC subtype signatures included Bailey progenitor and squamous programs and Moffitt classical and basal‐like programs, which are known to exhibit strong correspondence across bulk and single‐cell datasets.

As expected, established PDAC subtype signatures exhibited strong mutual correlations, including high concordance between the Bailey progenitor and Moffitt classical signatures, and between the Bailey squamous and Moffitt basal‐like signatures, confirming the internal consistency of the reference scoring framework. In contrast, the divergent‐edge modules did not show one‐to‐one correspondence with any individual subtype signature. The IGE module displayed a moderate positive correlation with the Bailey squamous signature, while the MPC module showed moderate anticorrelation with both classical subtype signatures. Beyond these limited associations, the remaining modules exhibited weak or negligible correlations with established subtype scores. Together, these results indicate that the divergent‐edge modules do not simply recapitulate expression‐defined PDAC subtypes, but instead capture regulatory programs that are largely orthogonal to existing classification schemes (Fig. 6).

Comparison with functional program signatures further clarified the biological specificity of individual modules. The IGE module showed a strong positive association with oxidative phosphorylation, whereas MPC was strongly anticorrelated with this program (Fig. 6). SAT displayed a moderate correlation with both the integrated stress response program and oxidative phosphorylation, while the IL2 module remained largely orthogonal to known functional programs. Together, these patterns indicate that the divergent‐edge modules reflect distinct adaptive biological processes—encompassing biosynthetic–metabolic activity, stress tolerance, immune evasion, and collective invasion—that intersect with but are not completely captured by any single established functional program.

### 3.7 Potential impact of clinical heterogeneity

Across transcriptional modules, treatment‐associated differences did not reach statistical significance, consistent with limited sample size. However, IGE module activity exhibited a moderate effect size favoring untreated tumors, consistent with suppression of growth‐associated transcriptional coordination under therapeutic pressure. Conversely, the IL2‐linked module showed a moderate effect in the opposite direction, with higher activity in treated tumors, aligning with immune or stress‐associated signaling induced by therapy (Table S4).

### 3.8 Functional and clinical correlates of adaptive modules

To determine whether the adaptive transcriptional modules identified by co‐expression divergence correspond to experimentally measurable vulnerabilities, we integrated module scores from PDAC cell lines in DepMap (release 24Q4) with CRISPR–Cas9 dependency profiles (Chronos) and compound‐response measurements from the PRISM Repurposing Primary Screen [23, 25, 34]. This analysis revealed mechanistically coherent and module‐specific patterns of selective dependency and therapeutic sensitivity.

**Fig. 7:**
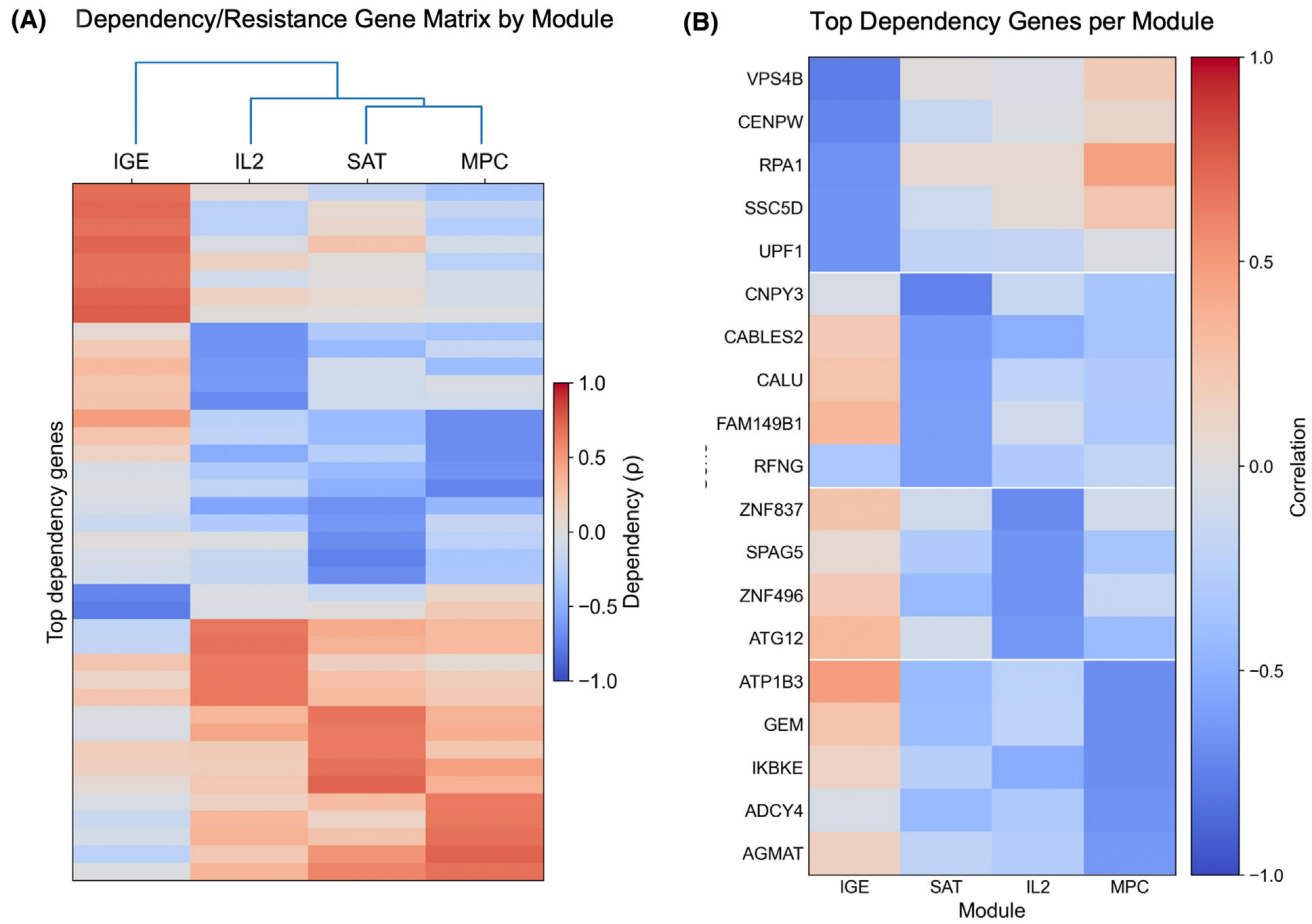
Dependency and resistance gene signatures across PDAC modules. (A) Heatmap showing the top dependency/resistance genes associated with each transcriptional module (IGE, IL2, SAT, MPC). For each module, the 10 genes with the strongest DepMap dependency correlations (|ρ|) were selected, combined across modules, and arranged by hierarchical clustering of modules (top dendrogram). Colors indicate correlation with dependency scores across PDAC cell lines (blue = higher dependency; red = resistance). (B) Gene‐resolved visualization of the top five module‐specific dependencies, organized by module. Horizontal dividers separate the 5‐gene sets chosen for each module. Colors represent correlation values (ρ) between gene dependency and module activity, highlighting distinct vulnerability profiles for each transcriptional program.

Notably, the ranked dependency matrix in Fig. 7A immediately reveals a two‐axis structure: the IGE‐associated dependencies form a tight, vertically aligned block enriched for biosynthetic and DNA‐repair factors, whereas the SAT, IL2, and MPC dependencies cluster together into a broader axis characterized by proteostasis, cytokine‐linked, and cytoskeletal regulators. The separation between these blocks is evident both in the color structure of the heatmap and in the hierarchical arrangement of modules in the top dendrogram, indicating that PDAC adaptation resolves into two orthogonal vulnerability regimes—a metabolic–biosynthetic IGE axis and a stress–immune–invasion axis comprising SAT, IL2, and MPC.

Across PDAC cell lines, the integrated growth‐energy (IGE) module exhibited strong dependencies on mitochondrial, ribosomal, and RNA‐processing genes—including *VPS4B*, *CENPW*, and *RPA1* (Fig. 7B)—consistent with a *MYC*‐ and *E2F*‐amplified biosynthetic state that relies on sustained translational output and oxidative phosphorylation [35, 36]. High‐IGE cell lines showed increased sensitivity to the PARP inhibitor talazoparib, the strongest association observed (ρ = −0.674, FDR = 0.065), coherent with replication‐stress and DNA‐repair liabilities inferred from IGE dependency signatures. Additional trend‐level correlations indicated sensitivity to inhibitors of PI3K/RTK signaling, highlighting the requirement for tightly regulated mitogenic and survival pathways in biosynthetically active states.

The stress‐adaptive transcription (SAT) module displayed a contrasting dependency profile, centered on ER‐resident folding and proteostasis regulators such as *CNPY3, CABLES2, CALU*, and *FAM149B1* (Fig. 7B). These features align with activation of the unfolded‐protein response (UPR) and integrated stress response (ISR) [37]. Drug‐response associations were generally modest but clustered around compounds that exacerbate proteotoxic load—including proteasome or cotranslational‐stress inhibitors—consistent with the prediction that ISR‐high tumor cells are vulnerable to forced proteostasis imbalance.

The IL2 module showed dependencies involving *ATG12, SPAG5*, and transcriptional regulators *ZNF837/ZNF496*, positioning the program at the interface of cytokine signaling, autophagy, and checkpoint‐associated survival (Fig. 7B). These patterns are coherent with the module's IL‐2/STAT5‐linked transcriptional identity and checkpoint‐aligned features [38, 39]. Drug‐response correlations were heterogeneous but included trend‐level sensitivity to agents that perturb vesicular trafficking or impose cytokine‐receptor stress, reinforcing the module's signaling architecture.

The multi‐pathway collective invasion (MPC) module was defined by dependencies on *ATP1B3, GEM*, and *IKBKE*, implicating polarity regulation, cytoskeletal remodeling, and NF‐κB signaling in maintaining collective motility (Fig. 7B). MPC‐high lines exhibited trend‐level sensitization to kinase‐stress and proteostasis‐targeting compounds (e.g., MG‐132, CHIR‐98014, BI‐D1870), whereas multiple RTK inhibitors showed relative resistance—consistent with the extensive RTK‐linked feedback wiring observed in basal‐like and invasive PDAC states [40, 41].

Correlation of module activity scores with PRISM drug response revealed both shared and program‐specific sensitivity patterns across the four transcriptional modules (Table S5). Tumors and cell lines with high IGE activity showed selective sensitivity to DNA damage and genome‐maintenance perturbations, including PARP inhibitors (talazoparib, olaparib), topoisomerase II inhibitors (etoposide), and transcriptional or proteostatic stressors (indisulam, MG‐132), alongside relative resistance to ERBB‐family and PI3K pathway inhibitors. In contrast, SAT, MPC, and IL2 modules exhibited more overlapping drug associations, with relative resistance to DNA‐damaging agents but increased sensitivity to growth‐ and proliferation‐linked stresses, including receptor tyrosine kinase inhibitors and cell‐cycle or mitotic regulators. Several compounds showed concordant associations across multiple modules, consistent with overlapping stress and growth programs rather than module‐exclusive drug dependencies. Together, these results indicate that divergent transcriptional programs encode distinct but partially overlapping therapeutic vulnerability landscapes, with IGE defining a stress‐adapted, genome‐instability–linked state that is pharmacologically separable from growth‐factor‐dependent programs.

**Fig. 8:**
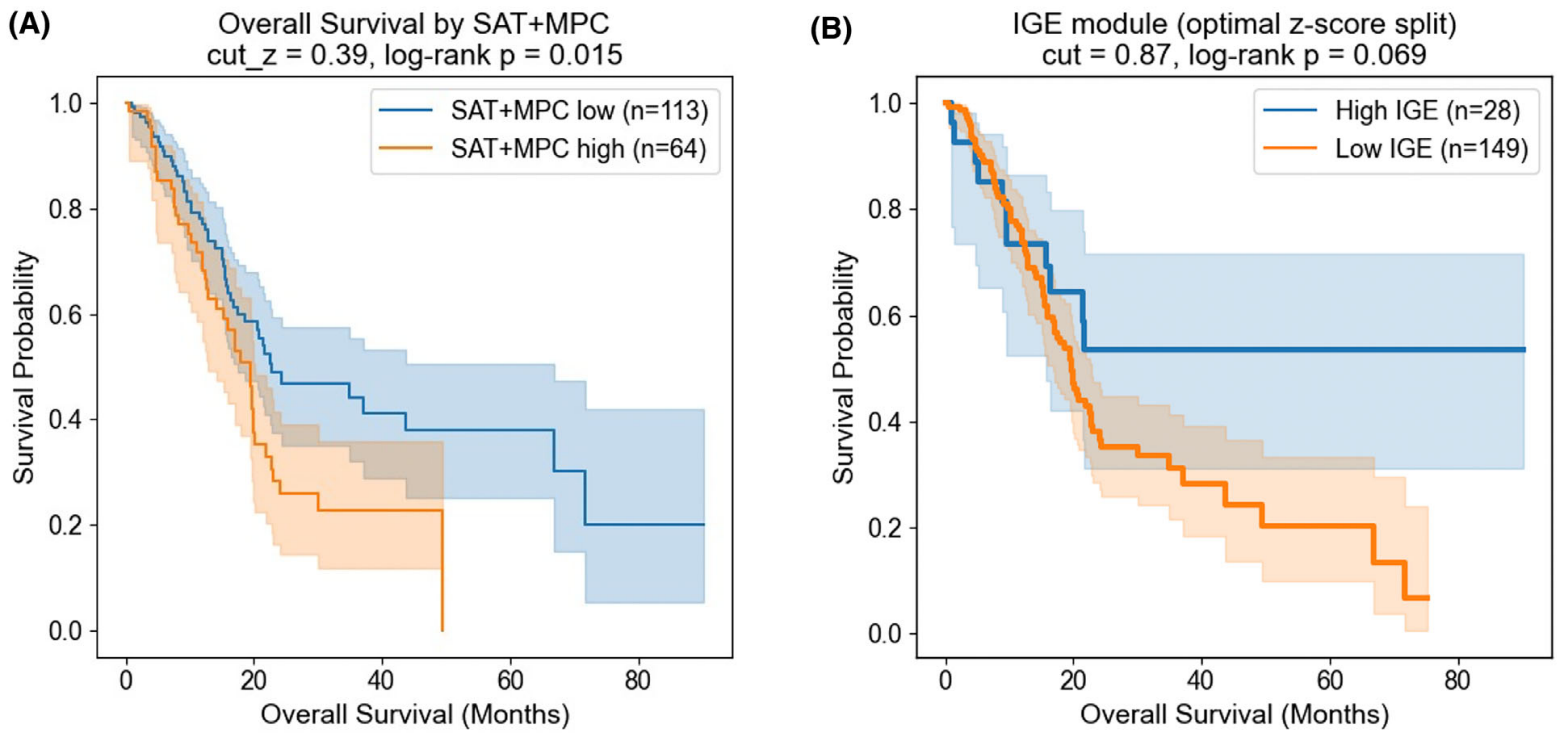
Survival impact of individual and combined transcriptional modules in PDAC. (A) Kaplan–Meier curves for the combined module score, generated by averaging the standardized SAT and MPC module activities and identifying an optimal split (cut = 0.80). Tumors with high combined module activity exhibit significantly poorer overall survival relative to the low‐score group (log‐rank *P* = 0.015; Mantel–Cox test), indicating that aggregate activation of the two PDAC programs captures a clinically meaningful risk state. (B) Kaplan–Meier analysis of the IGE module using an unbiased optimal *z*‐score cut point. A small subset of tumors with high IGE activity (*z* ≥ 0.87) shows markedly prolonged overall survival compared with the remainder of the cohort (log‐rank *P* = 0.069; Mantel–Cox test). This right‐tail subgroup corresponds to tumors with strong activation of the IGE transcriptional program. Kaplan–Meier analyses are shown for illustrative survival stratification, while multivariable Cox proportional hazards modeling adjusted for clinicopathologic covariates is reported separately (Table S5).

Finally, analysis of clinical cohorts demonstrated that module activity also bears prognostic significance (Fig. 8). When applied to bulk RNA‐seq data, ssGSEA scores reflect the average activity of each module across all cells within a tumor biopsy and may therefore attenuate signals arising from rare but transcriptionally distinct cell populations resolved in the single‐cell analyses. Despite this averaging, certain adaptive programs retained detectable associations with patient outcome, indicating that these programs are sufficiently prevalent to influence bulk tumor behavior.

Kaplan–Meier analyses were used to compare relative survival stratification across adaptive modules and their combinations in the TCGA‐PAAD cohort. Module activity scores were *z*‐scored across tumors and stratified using optimal cut points selected to maximize the log‐rank statistic, subject to a minimum group proportion constraint. These analyses are intended to illustrate relative survival stratification and are not designed to establish prognostic independence from clinicopathologic variables.

Among individual modules, SAT activity showed a trend toward poorer survival at high activation, consistent with prior work linking stress‐adaptive and proteostasis programs to aggressive tumor behavior. MPC activity alone did not significantly stratify survival. However, combined SAT and MPC activity produced the strongest and most robust survival separation, identifying a dominant stress–invasion fitness axis detectable in bulk transcriptomic data (Fig. 8A; Table S6).

In contrast, IGE activity did not significantly stratify survival overall but exhibited a favorable right‐tail effect at high activation, consistent with reports that classical, OXPHOS‐enriched PDAC shows moderately better prognosis than basal‐like disease (Fig. 8B). IL2 activity did not stratify survival in bulk cohorts, likely reflecting its confinement to a minority malignant subpopulation and the requirement for high immune infiltration—heterogeneous across TCGA tumors—for IL2‐linked immune evasion programs to manifest clinically. Although IL2 activity did not independently stratify clinical outcome, its functional coupling to SAT and MPC in single‐cell and functional dependency analyses places it within a broader adaptive stress–immune–invasion landscape.

To assess whether observed survival associations were independent of established clinicopathologic factors, we performed multivariable Cox proportional hazards modeling incorporating AJCC pathologic stage and tumor grade. In these models, adaptive module scores did not retain independent prognostic significance after adjustment, whereas pathologic stage remained the dominant predictor of overall survival (Table S7).

A limitation of the current study is that we did not directly address whether systemic therapy and metastatic dissemination can influence the adaptive modules. In the present study, network inference was dominated by treatment‐naive primary tumors, with only four post‐therapy samples included, limiting the ability to assess therapy‐ or site‐specific effects. Larger longitudinal and site‐matched cohorts will be required to explicitly resolve treatment‐ and metastasis‐associated network remodeling.

Together, these analyses indicate that the balance among biosynthetic, stress‐adaptive, immune‐modulatory, and invasive programs shapes tumor fitness and clinical behavior. The convergence of patterns observed across DepMap dependency data, PRISM drug‐sensitivity screens, and survival analyses supports the biological coherence of these adaptive modules and underscores their utility as interpretable representations of PDAC fitness states.

## 4 Discussion

This study shows that transcriptional heterogeneity in pancreatic ductal adenocarcinoma (PDAC) is driven not only by differences in gene expression levels but by systematic rewiring of gene–gene coordination across malignant cell states. By quantifying divergence in co‐expression rather than abundance, we identified four recurrent adaptive modules—biosynthetic–metabolic, stress‐adaptive, IL‐2–linked immune evasion, and collective invasion—that capture major axes of PDAC cell‐state plasticity. Unlike conventional clustering‐based approaches, which treat transcriptional states as discrete endpoints, our framework uses Leiden clusters only as scaffolds to expose how regulatory relationships shift across contexts. The key innovation lies in the divergent‐edge strategy itself: measuring how correlation patterns fracture and reassemble across tumor subpopulations, thereby revealing dynamic regulatory modules that are invisible to static expression or network‐average analyses. The resulting modules, although aligned with states described in recent single‐cell and spatial PDAC atlases [5, 6, 7, 8, 9, 10, 11, 12] emerge here from unbiased network‐level behavior rather than predefined biological categories.

A central conceptual advance of this work is that integrating these divergence‐derived modules with functional genomic data fundamentally reorganizes their interpretation. While the modules initially appear as four parallel adaptive states, their selective vulnerabilities collapse them into two higher‐order adaptive axes when viewed through the lens of CRISPR–Cas9 gene dependencies and drug‐response correlations. The biosynthetic–metabolic module (IGE) forms one axis, marked by reliance on mitochondrial, ribosomal, and RNA‐processing machinery, consistent with MYC–E2F‐driven anabolic programs. In contrast, the stress‐adaptive, IL‐2 immune evasion, and collective invasion modules converge into a second axis defined by proteostasis, cytokine‐linked signaling, membrane trafficking, and cytoskeletal dependencies. This reorganization—revealed only by integrating DepMap Chronos and PRISM response data—shows that PDAC cell states do not represent independent phenotypic islands, but instead reflect two overarching adaptive strategies: one that maximizes proliferative and metabolic efficiency, and another that orchestrates stress tolerance, immune escape, and cooperative invasion. The divergent‐edge modules identified here are not intended to define new PDAC subtypes. Rather, they represent adaptive biological processes that can be variably engaged by malignant cells, may coexist within individual tumors, and may change over time, consistent with the well‐established plasticity of PDAC cell states. These higher‐order adaptive strategies parallel emerging views of PDAC biology in which transcriptional state transitions are tightly coupled to metabolic specialization and microenvironmental constraints [13, 14, 15]. This interpretation is consistent with recent syntheses emphasizing that metabolic and stress‐adaptive programs jointly shape PDAC progression and therapy response [13, 14, 15].

Notably, these functional axes were not detectable from expression profiles, GO enrichment, or network topology alone. Importantly, these adaptive processes are not simple re‐descriptions of previously defined pathways. Because the modules are defined by coordinated behavior rather than by lists of upregulated genes, they incorporate both activating and restraining components. For example, the IGE process includes coordinated negative regulators such as *NRG4* and *CHD9* alongside biosynthetic and metabolic programs, features not captured by canonical oxidative phosphorylation or MYC‐associated signatures. The dependency and drug‐response signatures expose latent connections: for example, the shared reliance of SAT, IL2, and MPC on proteostasis and trafficking machinery; or the selective sensitization of IGE‐high lines to PARP inhibition and related DNA‐repair stressors. These patterns transform the modules from descriptive co‐expression communities into mechanistically grounded adaptive circuits with immediate translational relevance. They suggest that targeting PDAC requires different therapeutic strategies depending on whether tumors are aligned with a growth‐energy axis or a stress–immune–invasion axis, and that combinations should be rationally chosen to exploit the specific selective vulnerabilities of each adaptive strategy. Consistent with this process‐level interpretation, individual modules exhibit distinct biological specificities that extend beyond existing functional annotations. The IL2 module uniquely incorporates IL2 and receptor‐associated components, distinguishing it from generic immune evasion signatures. The SAT module spans a broader stress‐adaptive response than canonical integrated stress response programs, while the MPC module reflects coordinated collective invasion rather than single‐cell motility or epithelial–mesenchymal transition alone.

The clinical associations observed across TCGA‐PAAD further support this logic. SAT and MPC activities were associated with poorer survival, consistent with stress‐adapted and invasive phenotypes documented in prior PDAC studies [5, 7, 10, 12]. IGE showed a modest trend toward improved outcomes, paralleling classical‐like metabolic states, while the IL2 module displayed no dominant prognostic effect—possibly reflecting its selective use in immunologically insulated niches. Taken together, these findings indicate that the interplay among biosynthetic, stress‐protective, immune‐evasive, and migratory programs shapes both tumor progression and therapeutic vulnerability. The lack of independence of adaptive module scores in multivariable models is biologically consistent with this interpretation. These programs represent regulatory states that are mechanistically upstream of clinical disease stage rather than independent prognostic biomarkers. Accordingly, they define high‐risk adaptive tumor states whose clinical impact is reflected in advanced pathologic stage.

These observations highlight that prognostic effects in PDAC depend not only on transcriptional state labels but on the deeper regulatory strategies that underlie them. In this context, the role of *MYC* deserves particular nuance. Although *MYC* activation is frequently associated with aggressive biology in PDAC [35, 36], our findings suggest that its impact is highly contingent on regulatory context rather than uniformly deleterious.

The IGE module—defined by *MYC–E2F*–driven ribosomal and mitochondrial programs—did not associate with poorer survival and, in fact, displayed a small IGE‐high subgroup with modestly improved survival. This pattern aligns with recent work showing that classical or OXPHOS‐enriched PDAC subtypes, which often exhibit moderate *MYC* activity, can have relatively favorable clinical behavior compared with basal‐like states [5, 40]. In these settings, *MYC*‐driven biosynthesis appears to remain coupled to retained epithelial differentiation and metabolic coherence, rather than to dedifferentiation or cell‐cycle deregulation.

Thus, *MYC* overexpression in PDAC should not be interpreted as uniformly adverse. Its prognostic impact depends on whether *MYC*‐mediated anabolic programs operate within a stable epithelial‐like state—as in the IGE axis—or become integrated into stress‐adaptive or invasive phenotypes, where *MYC* cooperates with broader transcriptional instability. This contextual dependence further supports the view that adaptive modules, rather than individual transcription factors alone, shape the clinical trajectories of PDAC tumors.

The framework also may extend beyond PDAC. Divergent‐edge analysis, by focusing on regulatory reconfiguration, is likely to identify adaptive modules in other plastic malignancies and developmental systems. In preliminary analyses of pancreatic endocrine development, the same approach highlighted modules governing progenitor maintenance and β‐cell differentiation, suggesting shared regulatory logic between developmental transitions and malignant adaptation. More broadly, quantifying context‐specific shifts in gene coordination may capture a general principle by which cells restructure regulatory networks in response to selection pressures.

This study has several limitations. Divergent edges capture inferred shifts in transcriptional coordination rather than direct regulatory interactions, and although ALRA imputation reduces dropout‐related noise, residual technical biases may still influence correlation structure. Integration with DepMap and PRISM is constrained by the limited diversity of available PDAC cell lines, which may not fully reflect the heterogeneity of patient tumors. Establishing causality will require experimental perturbation of predicted regulatory nodes—such as *MYC* and *E2F1* in the biosynthetic program, *ATF4–DDIT3* in the stress‐adaptive axis, *JAK3/STAT5* in IL‐2–linked immune evasion, and EGFR/Rho/TGF‐β components in collective invasion.

Despite these constraints, coupling divergent‐edge networks with functional genomics reveals a coherent architecture of PDAC plasticity. By grounding transcriptional modules in experimentally defined dependencies, this framework moves beyond descriptive cell‐state classification toward a mechanistic understanding of how PDAC adapts, survives, and resists therapy. The integration of co‐expression divergence with gene dependency and drug–response data demonstrates that dynamic regulatory rewiring is not merely a transcriptional phenomenon but a predictor of selective vulnerability.

Future work should test whether the four modules function as causal adaptive circuits or represent the convergence of multiple upstream pressures. Spatial transcriptomics, multiplexed proteomics, and chromatin accessibility profiling will clarify how these states are arranged within the tumor microenvironment and whether the SAT–IL2–MPC axis forms discrete niches or a continuous adaptive gradient. Expanding dependency mapping to organoids, patient‐derived xenografts, and longitudinal treatment datasets may reveal how these modules shift under therapeutic pressure. More broadly, applying divergent‐edge analysis to other plastic malignancies—or to developmental and regenerative processes—may determine whether the two‐axis structure uncovered here reflects a general principle of cellular adaptation.

## 5 Conclusions

This study demonstrates that transcriptional plasticity in pancreatic ductal adenocarcinoma is driven not only by changes in gene expression levels but by systematic rewiring of gene–gene coordination across malignant cell states. By quantifying co‐expression divergence, we identified four adaptive transcriptional modules—IGE, SAT, IL2, and MPC—that capture major regulatory programs underlying biosynthetic activation, stress tolerance, immune evasion, and collective invasion. Integrating these modules with CRISPR dependency and PRISM drug‐response data revealed that they collapse into two orthogonal vulnerability axes: a biosynthetic–metabolic axis marked by MYC/E2F‐linked dependencies and selective sensitivity to PARP inhibition, and a stress–immune–invasion axis associated with proteostasis, cytokine, and cytoskeletal liabilities. These axes also carry distinct clinical implications, with SAT and MPC associated with poorer survival and a small IGE‐high subgroup showing modestly improved prognosis. Together, these findings show that divergent‐edge network analysis exposes mechanistically grounded adaptive circuits in PDAC, offering a framework for rational therapeutic targeting based on the selective vulnerabilities that arise as tumors rewire their regulatory architecture.

## Conflict of interest

The authors declare no conflict of interest.

## Author contributions

TM and SP conceptualized and designed the study. BN performed data processing and conducted the majority of the computational analyses. SP conducted additional computational analyses and wrote the original draft. TI and GB analyzed data and contributed conceptual insights. LD‐C and SP assembled the tables. LD‐C, NM, and LFE‐H assisted with analysis of DepMap and PRISM datasets. AY assisted with analysis and provided discussions that contributed to conceptual clarity. KRS provided funding, resources, and supervision. All authors contributed to revising the manuscript and approved the final version.

## Supporting information


**Fig. S1.** Harmony integration reduces study‐ and sample‐specific batch effects among malignant cells.
**Fig. S2.** Distribution of Bailey PDAC subtype signature scores before and after Harmony integration.
**Fig. S3.** Size distribution of malignant Leiden clusters.
**Fig. S4.** Additional characterization of the MPC.
**Table S1.** Clinicopathologic characteristics of PDAC cohorts analyzed in this study.
**Table S2.** The number of cells and percentage of total cells for each Leiden cluster of malignant PDAC epithelial cells.
**Table S3.** Gene membership for divergent‐edge modules.
**Table S4.** Sample‐level statistical comparison of transcriptional module activity stratified by treatment status and anatomical site.
**Table S5.** Drug–module associations across transcriptional programs.
**Table S6.** Survival stratification by adaptive module activity in TCGA‐PAAD.
**Table S7.** Multivariable Cox proportional hazards analysis of transcriptional module activity in PDAC.

## Data Availability

Single‐cell RNA‐seq data were obtained from two published pancreatic ductal adenocarcinoma (PDAC) datasets: Peng et al. (CRA001160) and Werba et al. (GSM6204109) [12, 26]. For the Peng dataset, raw fastq files were downloaded from the Genome Sequence Archive. For the Werba dataset, BAM files were retrieved from GEO and converted to fastq using bamtofastq (10× Genomics). All single‐cell count matrices and downstream analyses were generated from these publicly available resources. DepMap gene dependency (Chronos) data, CCLE transcriptomic profiles, and PRISM drug‐response matrices were downloaded from the DepMap portal (https://depmap.org). Scripts used in the analysis are available at: https://github.com/RScottPowers‐genome/Network‐Divergence‐Analysis‐PDAC.

## References

[1] Groves SM , Quaranta V . Quantifying cancer cell plasticity with gene regulatory networks and single‐cell dynamics. Front Netw Physiol. 2023;3:1225736. 37731743 10.3389/fnetp.2023.1225736 PMC10507267

[2] Bhat GR , Sethi I , Sadida HQ , Rah B , Mir R , Algehainy N , et al. Cancer cell plasticity: from cellular, molecular, and genetic mechanisms to tumor heterogeneity and drug resistance. Cancer Metastasis Rev. 2024;43:197–228. 38329598 10.1007/s10555-024-10172-z PMC11016008

[3] Cordani M , Dando I , Ambrosini G , Gonzalez‐Menendez P . Signaling, cancer cell plasticity, and intratumor heterogeneity. Cell Commun Signal. 2024;22:255. 38702718 10.1186/s12964-024-01643-5 PMC11067149

[4] Huang S , Soto AM , Sonnenschein C . The end of the genetic paradigm of cancer. PLoS Biol. 2025;23:e3003052. 40100793 10.1371/journal.pbio.3003052 PMC12136056

[5] Pitter KL , Grbovic‐Huezo O , Joost S , Singhal A , Blum M , Wu K , et al. Systematic comparison of pancreatic ductal adenocarcinoma models identifies a conserved highly plastic basal cell state. Cancer Res. 2022;82:3549–3560. 35952360 10.1158/0008-5472.CAN-22-1742 PMC9532381

[6] Raghavan S , Winter PS , Navia AW , Williams HL , DenAdel A , Lowder KE , et al. Microenvironment drives cell state, plasticity, and drug response in pancreatic cancer. Cell. 2021;184:6119–6137.e26. 34890551 10.1016/j.cell.2021.11.017 PMC8822455

[7] Barthel S , Falcomata C , Rad R , Theis FJ , Saur D . Single‐cell profiling to explore pancreatic cancer heterogeneity, plasticity and response to therapy. Nat Cancer. 2023;4:454–467. 36959420 10.1038/s43018-023-00526-x PMC7615362

[8] Grunwald BT , Devisme A , Andrieux G , Vyas F , Aliar K , McCloskey CW , et al. Spatially confined sub‐tumor microenvironments in pancreatic cancer. Cell. 2021;184:5577–5592.e18. 34644529 10.1016/j.cell.2021.09.022

[9] Steele NG , Carpenter ES , Kemp SB , Sirihorachai VR , The S , Delrosario L , et al. Multimodal mapping of the tumor and peripheral blood immune landscape in human pancreatic cancer. Nat Cancer. 2020;1:1097–1112. 34296197 10.1038/s43018-020-00121-4 PMC8294470

[10] Williams HL , Dias Costa A , Zhang J , Raghavan S , Winter PS , Kapner KS , et al. Spatially resolved single‐cell assessment of pancreatic cancer expression subtypes reveals co‐expressor phenotypes and extensive intratumoral heterogeneity. Cancer Res. 2023;83:441–455. 36459568 10.1158/0008-5472.CAN-22-3050 PMC10548885

[11] Park JK , Jeong HO , Kim H , Choi JH , Lee EM , Kim S , et al. Single‐cell transcriptome analysis reveals subtype‐specific clonal evolution and microenvironmental changes in liver metastasis of pancreatic adenocarcinoma and their clinical implications. Mol Cancer. 2024;23:87. 38702773 10.1186/s12943-024-02003-0 PMC11067162

[12] Werba G , Weissinger D , Kawaler EA , Zhao E , Kalfakakou D , Dhara S , et al. Single‐cell RNA sequencing reveals the effects of chemotherapy on human pancreatic adenocarcinoma and its tumor microenvironment. Nat Commun. 2023;14:797. 36781852 10.1038/s41467-023-36296-4 PMC9925748

[13] Espinet E , Klein L , Pure E , Singh SK . Mechanisms of PDAC subtype heterogeneity and therapy response. Trends Cancer. 2022;8:1060–1071. 36117109 10.1016/j.trecan.2022.08.005

[14] Mehla K , Singh PK . Metabolic subtyping for novel personalized therapies against pancreatic cancer. Clin Cancer Res. 2020;26:6–8. 31628144 10.1158/1078-0432.CCR-19-2926 PMC6942627

[15] Ohara Y , Liu H , Moreno P , Suzuki S , Hussain SP . Molecular, metabolic, and histological subtypes of pancreatic ductal adenocarcinoma and its tumor microenvironment: insights into tumor heterogeneity and clinical implications. Pharmacol Ther. 2026;277:108946. 41183744 10.1016/j.pharmthera.2025.108946

[16] Nieto MA , Huang RY , Jackson RA , Thiery JP . Emt: 2016. Cell. 2016;166:21–45. 27368099 10.1016/j.cell.2016.06.028

[17] Quintanal‐Villalonga A , Chan JM , Yu HA , Pe'er D , Sawyers CL , Sen T , et al. Lineage plasticity in cancer: a shared pathway of therapeutic resistance. Nat Rev Clin Oncol. 2020;17:360–371. 32152485 10.1038/s41571-020-0340-z PMC7397755

[18] Spranger S , Bao R , Gajewski TF . Melanoma‐intrinsic beta‐catenin signalling prevents anti‐tumour immunity. Nature. 2015;523:231–235. 25970248 10.1038/nature14404

[19] Kiselev VY , Andrews TS , Hemberg M . Challenges in unsupervised clustering of single‐cell RNA‐seq data. Nat Rev Genet. 2019;20:273–282. 30617341 10.1038/s41576-018-0088-9

[20] Pratapa A , Jalihal AP , Law JN , Bharadwaj A , Murali TM . Benchmarking algorithms for gene regulatory network inference from single‐cell transcriptomic data. Nat Methods. 2020;17:147–154. 31907445 10.1038/s41592-019-0690-6 PMC7098173

[21] Saint‐Antoine MM , Singh A . Network inference in systems biology: recent developments, challenges, and applications. Curr Opin Biotechnol. 2020;63:89–98. 31927423 10.1016/j.copbio.2019.12.002 PMC7308210

[22] Larsson I , Held F , Popova G , Koc A , Kundu S , Jornsten R , et al. Reconstructing the regulatory programs underlying the phenotypic plasticity of neural cancers. Nat Commun. 2024;15:9699. 39516198 10.1038/s41467-024-53954-3 PMC11549355

[23] Meyers RM , Bryan JG , McFarland JM , Weir BA , Sizemore AE , Xu H , et al. Computational correction of copy number effect improves specificity of CRISPR‐Cas9 essentiality screens in cancer cells. Nat Genet. 2017;49:1779–1784. 29083409 10.1038/ng.3984 PMC5709193

[24] Pacini C , Dempster JM , Boyle I , Goncalves E , Najgebauer H , Karakoc E , et al. Integrated cross‐study datasets of genetic dependencies in cancer. Nat Commun. 2021;12:1661. 33712601 10.1038/s41467-021-21898-7 PMC7955067

[25] Tsherniak A , Vazquez F , Montgomery PG , Weir BA , Kryukov G , Cowley GS , et al. Defining a cancer dependency map. Cell. 2017;170:564–576.e16. 28753430 10.1016/j.cell.2017.06.010 PMC5667678

[26] Peng J , Sun BF , Chen CY , Zhou JY , Chen YS , Chen H , et al. Single‐cell RNA‐seq highlights intra‐tumoral heterogeneity and malignant progression in pancreatic ductal adenocarcinoma. Cell Res. 2019;29:725–738. 31273297 10.1038/s41422-019-0195-y PMC6796938

[27] Fleming SJ , Chaffin MD , Arduini A , Akkad AD , Banks E , Marioni JC , et al. Unsupervised removal of systematic background noise from droplet‐based single‐cell experiments using CellBender. Nat Methods. 2023;20:1323–1335. 37550580 10.1038/s41592-023-01943-7

[28] Wolock SL , Lopez R , Klein AM . Scrublet: computational identification of cell doublets in single‐cell transcriptomic data. Cell Syst. 2019;8:281–291.e9. 30954476 10.1016/j.cels.2018.11.005 PMC6625319

[29] Wolf FA , Angerer P , Theis FJ . SCANPY: large‐scale single‐cell gene expression data analysis. Genome Biol. 2018;19:15. 29409532 10.1186/s13059-017-1382-0 PMC5802054

[30] Patel AP , Tirosh I , Trombetta JJ , Shalek AK , Gillespie SM , Wakimoto H , et al. Single‐cell RNA‐seq highlights intratumoral heterogeneity in primary glioblastoma. Science. 2014;344:1396–1401. 24925914 10.1126/science.1254257 PMC4123637

[31] Korsunsky I , Millard N , Fan J , Slowikowski K , Zhang F , Wei K , et al. Fast, sensitive and accurate integration of single‐cell data with harmony. Nat Methods. 2019;16:1289–1296. 31740819 10.1038/s41592-019-0619-0 PMC6884693

[32] Linderman GC , Zhao J , Roulis M , Bielecki P , Flavell RA , Nadler B , et al. Zero‐preserving imputation of single‐cell RNA‐seq data. Nat Commun. 2022;13:192. 35017482 10.1038/s41467-021-27729-z PMC8752663

[33] Mittal D , Lepletier A , Madore J , Aguilera AR , Stannard K , Blake SJ , et al. CD96 is an immune checkpoint that regulates CD8(+) T‐cell antitumor function. Cancer Immunol Res. 2019;7:559–571. 30894377 10.1158/2326-6066.CIR-18-0637 PMC6445751

[34] Corsello SM , Bittker JA , Liu Z , Gould J , McCarren P , Hirschman JE , et al. The drug repurposing hub: a next‐generation drug library and information resource. Nat Med. 2017;23:405–408. 28388612 10.1038/nm.4306 PMC5568558

[35] Bailey P , Chang DK , Nones K , Johns AL , Patch AM , Gingras MC , et al. Genomic analyses identify molecular subtypes of pancreatic cancer. Nature. 2016;531:47–52. 26909576 10.1038/nature16965

[36] Ying H , Dey P , Yao W , Kimmelman AC , Draetta GF , Maitra A , et al. Genetics and biology of pancreatic ductal adenocarcinoma. Genes Dev. 2016;30:355–385. 26883357 10.1101/gad.275776.115 PMC4762423

[37] Pakos‐Zebrucka K , Koryga I , Mnich K , Ljujic M , Samali A , Gorman AM . The integrated stress response. EMBO Rep. 2016;17:1374–1395. 27629041 10.15252/embr.201642195 PMC5048378

[38] LaFleur MW , Muroyama Y , Drake CG , Sharpe AH . Inhibitors of the PD‐1 pathway in tumor therapy. J Immunol. 2018;200:375–383. 29311378 10.4049/jimmunol.1701044 PMC5924692

[39] Mlecnik B , Bindea G , Angell HK , Maby P , Angelova M , Tougeron D , et al. Integrative analyses of colorectal cancer show immunoscore is a stronger predictor of patient survival than microsatellite instability. Immunity. 2016;44:698–711. 26982367 10.1016/j.immuni.2016.02.025

[40] Chan‐Seng‐Yue M , Kim JC , Wilson GW , Ng K , Figueroa EF , O'Kane GM , et al. Transcription phenotypes of pancreatic cancer are driven by genomic events during tumor evolution. Nat Genet. 2020;52:231–240. 31932696 10.1038/s41588-019-0566-9

[41] Puleo F , Nicolle R , Blum Y , Cros J , Marisa L , Demetter P , et al. Stratification of pancreatic ductal adenocarcinomas based on tumor and microenvironment features. Gastroenterology. 2018;155:1999–2013.e3. 30165049 10.1053/j.gastro.2018.08.033

