# Supplementary material for: Network divergence analysis identifies adaptive gene modules and two orthogonal vulnerability axes in pancreatic cancer
Source: Mol Oncol. 2026 Mar 1;20(7):1857–76. doi: 10.1002/1878-0261.70218 (PMC13352960; doi:10.1002/1878-0261.70218)
Supplement: Supplementary file 1 — Fig. S1. Harmony integration reduces study‐ and sample‐specific batch effects among malignant cells. Fig. S2. Distribution of Bailey PDAC subtype signature scores before and after Harmony integration. Fig. S3. Size distribution of malignant Leiden clusters. Fig. S4. Additional characterization of the MPC. Table S1. Clinicopathologic characteristics of PDAC cohorts analyzed in this study. Table S2. The number of cells and percentage of total cells for each Leiden cluster of malignant PDAC epithelial cells. Table S3. Gene membership for divergent‐edge modules. Table S4. Sample‐level statistical comparison of transcriptional module activity stratified by treatment status and anatomical site. Table S5. Drug–module associations across transcriptional programs. Table S6. Survival stratification by adaptive module activity in TCGA‐PAAD. Table S7. Multivariable Cox proportional hazards analysis of transcriptional module activity in PDAC. [file MOL2-20-1857-s001.zip › mol270218-sup-0001-Supinfo.pdf]

Supplementary Information

Supplementary Figure 1. Harmony integration reduces study- and sample-specific batch effects among malignant cells. UMAP visualizations of malignant cells before and after Harmony integration. Top panels show cells colored by study of origin (Peng vs Werba), and bottom panels show cells colored by individual sample. Prior to integration (left), cells segregate strongly by study and sample, indicating substantial batch effects. After Harmony integration (right), cells from different studies and samples are well mixed across the embedding, consistent with effective batch correction. Importantly, post-Harmony embeddings do not form discrete clusters, reflecting the continuous and plastic nature of malignant PDAC cell states rather than overcorrection into artificial groupings.

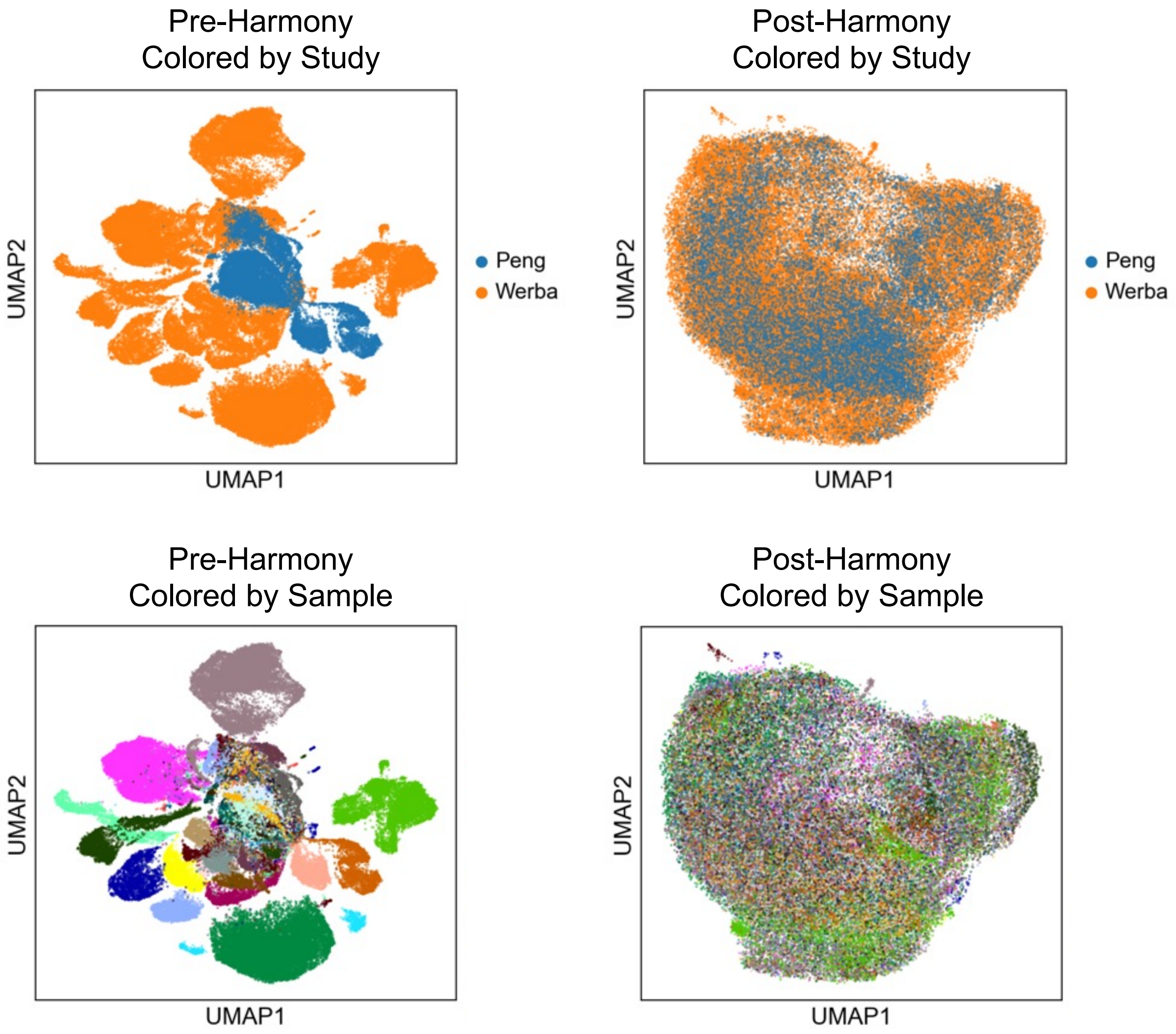

Supplementary Figure 2. Distribution of Bailey PDAC subtype signature scores before and after harmony integration. UMAP visualizations of malignant cells colored by Bailey subtype signature scores, including progenitor, squamous, ADEX, and immunogenic programs. Top row shows subtype scores projected onto the pre-Harmony embedding, and bottom row shows the same scores projected onto the post-Harmony embedding. Signature scores are displayed as continuous values, with higher scores indicated by warmer colors. Prior to integration, subtype-associated signal is partially confounded by study- and sample-specific structure. After Harmony integration, subtype signatures exhibit smoother, more continuous gradients across the malignant cell manifold, consistent with PDAC cell-state plasticity rather than discrete subtype boundaries. These patterns indicate that Harmony integration reduces technical batch effects while preserving biologically meaningful transcriptional programs.

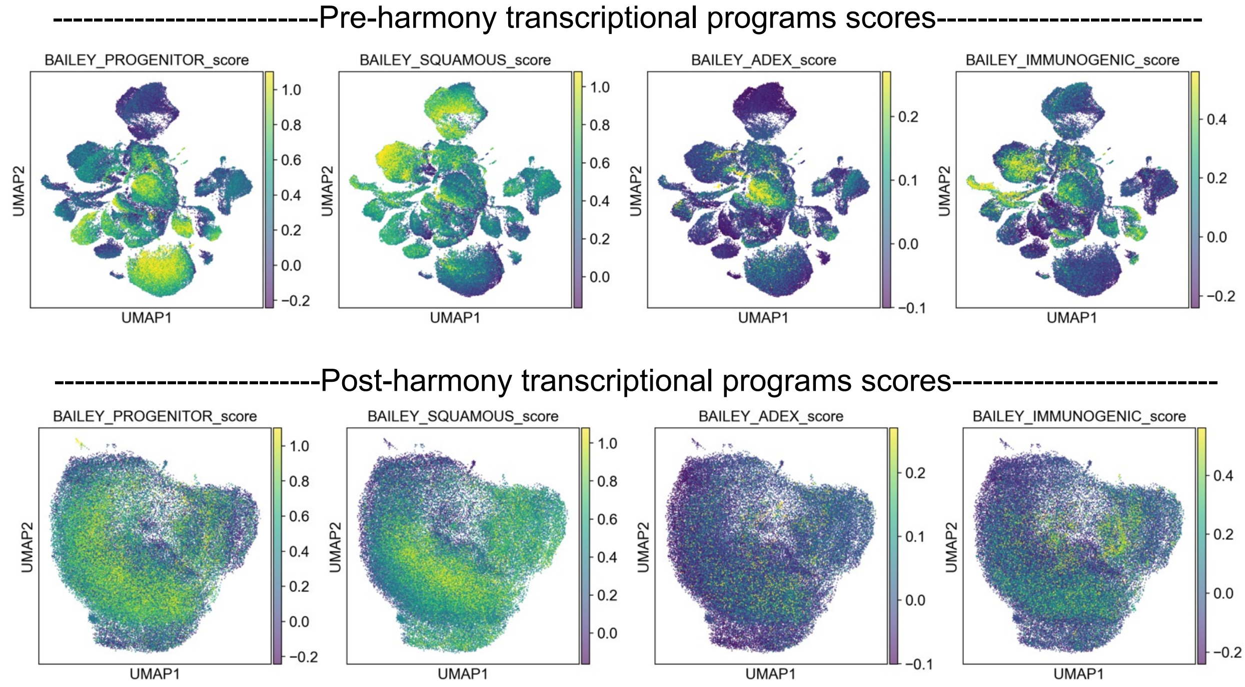

Supplementary Figure 3. Size distribution of malignant leiden clusters. Complementary cumulative distribution function (CCDF) of malignant Leiden cluster sizes following Harmony integration and clustering. Each point represents the probability that a randomly selected cluster contains at least k cells ($P(K \geq k)$), plotted as a function of cluster size on a log–log scale. The distribution illustrates the presence of a small number of large malignant clusters alongside a long tail of smaller clusters. Based on this distribution, downstream divergent-edge analyses focused on the ten largest clusters, which together account for approximately 70% of malignant cells and provide sufficient sample sizes for stable estimation of gene–gene correlations.

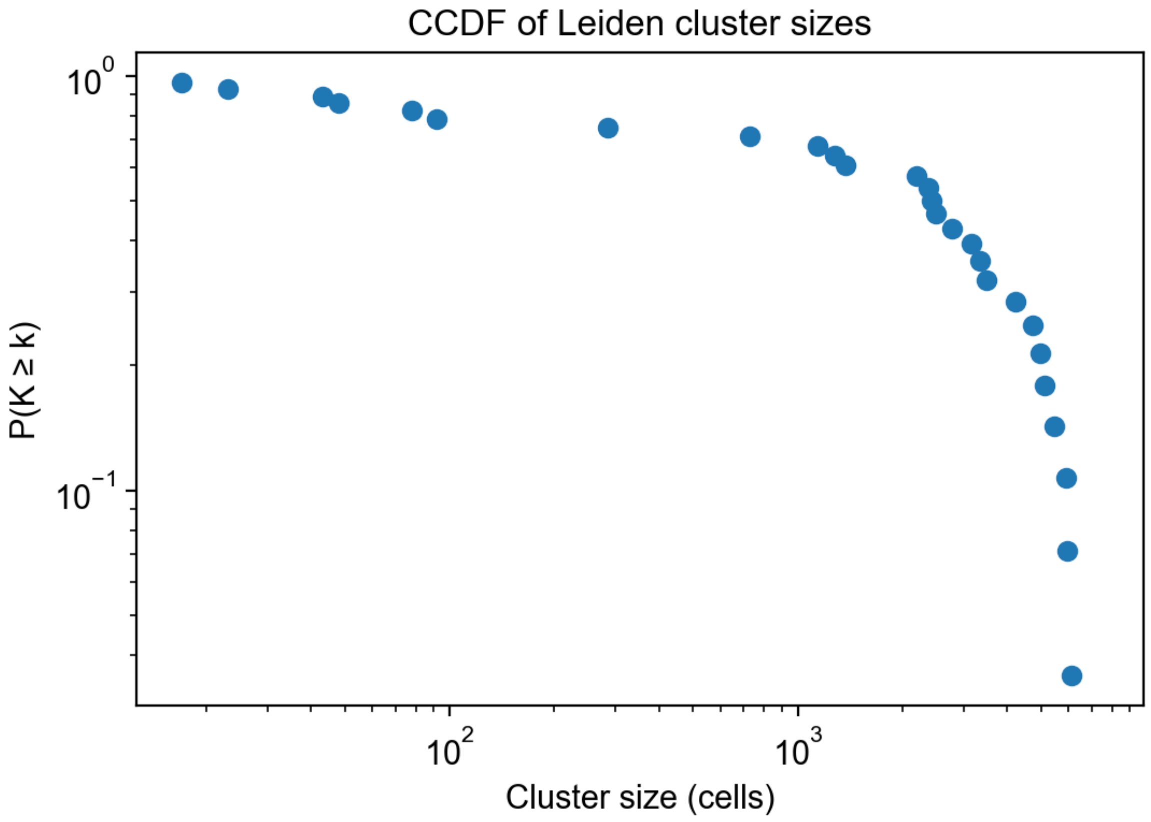

Supplementary Figure 4. Additional characterization of the MPC. (A) Network graph showing the interaction between core module signaling components (blue), membrane trafficking components (yellow), and Rab-regulators of membrane trafficking (orange), highlighting the role of vesicle transport in migration and invasion. (B) Core genes are shown in blue, while EGFR=ligand loop genes are highlighted in orange, illustrating their coordinated expression within the migration and invasion module

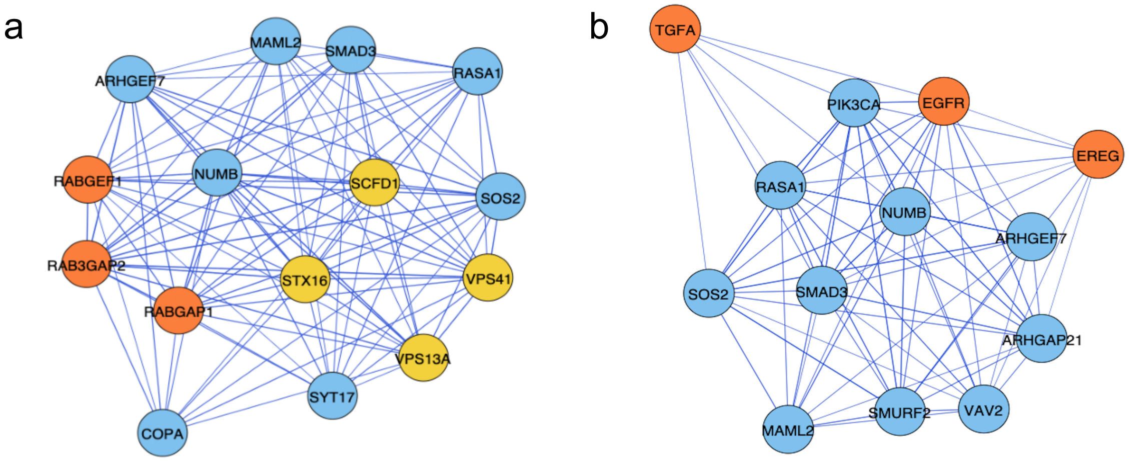

Supplementary Table 1. Clinicopathologic characteristics of PDAC cohorts analyzed in this study.

	Werba et al.	Peng et al.
**Total Number of cases included**	20	22
**Age at diagnosis, mean ± SD, y**	69.2 ± 9.11	60.6 ± 9.08
**Sex, No. (%)**		
Male	11 (55%)	10
Female	9 (45%)	12
**Distant metastasis: No. (%)**		
Yes	12 (60%)	-
No	8 (40%)	-
**Treatment before tissue collection: No. (%)**		
Yes	4 (20%)	-
No	16 (80%)	-
**Histologic grade (G), No. (%)**		
Poorly differentiated adenocarcinoma	5 (25%)	-
Moderately to poorly differentiated adenocarcinoma	6 (25%)	-
Moderately differentiated adenocarcinoma	6 (30%)	-
Well to moderately differentiated adenocarcinoma	1 (5%)	-
Well differentiated adenocarcinoma	2 (10%)	-
**Source: No. (%)**		
Pancreas	11 (55%)	100 (100%)
Liver	9 (45%)	-
**Specimen type, No. (%)**		
Biopsy	14 (70%)	-
Resection	6 (30%)	22 (100%)
**Maximum Diameter, mean ± SD, mm**	-	36.3 ± 20.6
**TNM Classification: No. (%)**		
T1N0M0	-	1 (4.54%)
T1N1M0	-	2 (9.01%)
T1N2M0	-	1 (4.54%)
T2N0M0	-	8 (36.4%)
T2N1M0	-	4 (18.1%)
T3N0M0	-	1 (4.54%)
T3N1M0	-	3 (13.6%)
T3N2M0	-	1 (4.54%)
T4N2M0	-	1 (4.54%)
**Staging**		
IB	-	9 (40.1%)
IIA	-	2 (9.01%)
IIB	-	8 (36.4%)
III	-	3 (13.6)
**Perineural Invasion: No. (%)**		
Yes	-	16 (72.7%)
No	-	6 (27.3%)
**Vascular Invasion: No. (%)**		
Yes	-	12 (54.5%)
No	-	10 (45.5%)
**Peripancreatic Infiltarion: No. (%)**		
Yes	-	20 (90.9%)
No	-	2 (9.01%)

Clinicopathologic characteristics of PDAC cases from the Werba and Peng cohorts, including patient demographics, treatment status, tumor grade, specimen type, tumor size, TNM classification, pathologic stage, and histopathologic features. Values are shown as number (percentage); missing data were not reported in the source studies.

Supplementary Table 2. The number of cells and percentage of total cells for each Leiden cluster of malignant PDAC epithelial cells.

Cluster	Cells	Percentage
0	7274	9.43%
1	6130	7.95%
2	5938	7.70%
3	5921	7.67%
4	5460	7.08%
5	5113	6.63%
6	4964	6.43%
7	4754	6.16%
8	4230	5.48%
9	3500	4.54%
10	3347	4.34%
11	3149	4.08%
12	2775	3.60%
13	2495	3.23%
14	2431	3.15%
15	2381	3.09%
16	2197	2.85%
17	1369	1.77%
18	1274	1.65%
19	1139	1.48%
20	729	0.94%
21	284	0.37%
22	92	0.12%
23	78	0.10%
24	48	0.06%
25	43	0.06%
26	23	0.03%
27	17	0.02%

Supplementary Table 3. Gene membership for divergent-edge modules.

This is provided as an Excel file.

Genes are listed according to module assignment derived from Leiden clustering of the divergent-edge network. Module membership reflects network topology and patterns of gene–gene coordination rather than gene-level statistical testing; therefore, no p-values or adjusted significance metrics are associated with individual genes in this table. For each module, core genes and peripheral genes are listed separately.

Supplementary Table 4. Sample-level statistical comparison of transcriptional module activity stratified by treatment status and anatomical site.

	IGE: UT vs T	IGE: Prim vs Met	SAT: UT vs T	SAT: Prim vs Met	MPC: UT vs T	MPC: Prim vs Met	IL2: UT vs T	IL2: Prim vs Met
Mann–Whitney	0.33	0.69	0.93	0.69	0.66	0.53	0.33	1.00
Permutation	0.23	1.00	0.46	0.91	0.44	0.49	0.06	0.89
Cliff's delta	0.40	-0.15	0.07	-0.15	-0.20	0.22	-0.40	0.00

Module activity was summarized per tumor using median cell-level scores. Group differences (untreated vs treated; primary vs liver metastasis) were assessed using two-sided Mann–Whitney U tests, permutation testing (10,000 permutations), and Cliff’s delta effect sizes.

Supplementary Table 5. Drug–module associations across transcriptional programs.

Drug	Primary process	IGE ρ (p)	SAT ρ (p)	MPC ρ (p)	IL2 ρ (p)	Directionality summary
Talazoparib	DNA damage repair (PARP)	−0.67 (4.5e-5)	—	—	—	IGE↓
Olaparib	DNA damage repair (PARP)	−0.51 (0.004)	—	—	—	IGE↓
Etoposide / etoposide-phosphate	DNA damage (TOP2)	−0.54 (0.003)	—	+0.44 (0.016)	+0.49 (0.008)	IGE↓; MPC/IL2↑
Mitoxantrone	DNA damage (TOP2/intercalator)	—	—	+0.63 (0.001)	+0.45 (0.029)	MPC/IL2↑
Indisulam	Transcription / splicing stress	−0.61 (0.00035)	—	—	—	IGE↓
MG-132	Proteostasis (proteasome)	−0.44 (0.016)	−0.37 (0.043)	—	—	IGE↓; SAT↓
Dacomitinib	ERBB family RTK	+0.57 (0.001)	—	—	—	IGE↑
Afatinib	ERBB family RTK	+0.54 (0.003)	—	—	—	IGE↑
AST-1306	ERBB family RTK	+0.81 (0.0049)	—	—	—	IGE↑
BMS-690514	EGFR/VEGFR RTK	+0.54 (0.0023)	—	—	—	IGE↑
XL-647	EGFR/VEGFR RTK	+0.54 (0.0019)	—	—	—	IGE↑
Alpelisib	PI3K signaling	+0.55 (0.0021)	—	—	—	IGE↑
PI3K-IN-2	PI3K signaling	+0.50 (0.0049)	—	—	—	IGE↑
VE-822	ATR / replication stress	—	+0.55 (0.0018)	—	—	SAT↑
Len vat inib	VEGFR/FGFR RTK	—	+0.50 (0.005)	+0.59 (0.0006)	—	SAT/MPC↑
SCS	Nonspecific / library compound	—	+0.63 (0.0027)	+0.66 (0.0015)	+0.49 (0.029)	Broad↑
MK-0773	CNS / nonspecific (GABA-A)	—	+0.52 (0.0068)	+0.48 (0.014)	+0.56 (0.0027)	Broad↑
Nilutamide	Androgen receptor signaling	—	+0.41 (0.034)	+0.45 (0.017)	+0.46 (0.016)	Broad↑

Spearman correlation coefficients (ρ) between drug sensitivity and module scores for IGE, SAT, MPC, and IL2 programs across PDAC cell lines, with associated p-values shown in parentheses. Drugs are grouped by primary biological process or target pathway. Directionality summary indicates consistent patterns of sensitivity or resistance across modules. Negative ρ denotes increased sensitivity in module-high cell lines, whereas positive ρ denotes relative resistance in module-high cell lines. Values shown as "—" indicate that the association did not meet the predefined inclusion threshold in that module.

Supplementary Table 6. Survival stratification by adaptive module activity in TCGA-PAAD.

Predictor	Optimal cutpoint (z)	Log-rank p	N high	N low
SAT+MPC	0.39	0.015	64	113
SAT+IL2	0.33	0.073	71	106
SAT	0.45	0.100	55	122
IL2	0.69	0.229	34	143
SAT+MPC+IL2	0.95	0.249	27	150
MPC+IL2	-0.63	0.310	142	35
MPC	0.37	0.416	66	111

Kaplan–Meier analyses were performed using an "optimal z-score split" procedure in which each module score (or summed combination) was z-scored across tumors and the cutpoint was chosen to maximize the log-rank statistic, subject to a minimum group proportion of 0.15 (high group defined as $z \geq$ cut). Reported are the optimal cutpoint, log-rank p-value, and resulting group sizes.

Supplementary Table 7. Multivariable Cox proportional hazards analysis of transcriptional module activity in PDAC.

covariate	coef	HR	CI95_low	CI95_high	p
STAGE_II	0.72	2.05	1.09	3.86	0.03
GRADE_G3-4	0.29	1.34	0.89	2.01	0.17
IGE_Z	0.11	1.12	0.86	1.46	0.40
SAT_Z	0.09	1.10	0.88	1.37	0.42
IL2_Z	0.10	1.10	0.84	1.44	0.48
STAGE_III-IV	0.29	1.34	0.43	4.24	0.62
MPC_Z	0.00	1.00	0.76	1.31	1.00

Multivariable Cox models were fitted for overall survival including standardized transcriptional module scores (SAT, MPC, IL2, IGE; hazard ratios reported per one standard deviation increase), AJCC pathologic stage (collapsed to I, II, and III–IV), and tumor grade (collapsed to G1–2 vs G3–4; GX excluded). Pathologic stage remained the dominant predictor of outcome, whereas individual module activities did not retain independent prognostic significance after adjustment.
